# Data on the interexaminer variation of minutia markup on latent fingerprints

**DOI:** 10.1016/j.dib.2016.04.068

**Published:** 2016-05-04

**Authors:** Bradford T. Ulery, R. Austin Hicklin, Maria Antonia Roberts, JoAnn Buscaglia

**Affiliations:** a Noblis, Falls Church, VA, USA; b Latent Print Support Unit, Federal Bureau of Investigation Laboratory Division, Quantico, VA, USA; c Counterterrorism and Forensic Science Research Unit, Federal Bureau of Investigation Laboratory Division, Quantico, VA, USA

**Keywords:** Biometrics, Latent fingerprint examination, Fingermark, ACE-V, Repeatability, Reproducibility

## Abstract

The data in this article supports the research paper entitled “Interexaminer variation of minutia markup on latent fingerprints” [1]. The data in this article describes the variability in minutia markup during both analysis of the latents and comparison between latents and exemplars. The data was collected in the “White Box Latent Print Examiner Study,” in which each of 170 volunteer latent print examiners provided detailed markup documenting their examinations of latent-exemplar pairs of prints randomly assigned from a pool of 320 pairs. Each examiner examined 22 latent-exemplar pairs; an average of 12 examiners marked each latent.

**Specifications Table**

**Table:** 

Subject area	*Forensic science*
More specific subject area	*Latent fingerprints*
Type of data	*Tables, graphs, text descriptions*
How data was acquired	*Markup of latent fingerprints by latent print examiners under test conditions*
Data format	*Analyzed*
Experimental factors	*Feature types, locations, correspondences; local ridge clarity; examiner determinations*
Experimental features	*Automated clustering algorithms used to classify minutiae marked by multiple examiners as representing the same minutia*
Data source location	
Data accessibility	*Data are within this article*

**Value of the data**

• Latent print examiners often differ in the features they use in the analysis and comparison of fingerprints. This data provides a wealth of information on how markup varies among examiners, how this relates to the quality of the fingerprints and to examiners’ differing determinations.
• We provide this data in order to serve as a benchmark, to strengthen the community׳s understanding of the latent print examination process.
• This data provides greater visibility into the bases for examiners’ decisions, and increases the community׳s understanding of subjectivity in latent print examination.
• This data may assist the community in deciding how to improve operational procedures, training, and standardization.
• This data may be of particular interest for automated fingerprint identification systems, which rely on human markup of minutiae.

## Data

This paper presents tables and charts describing the variation in how minutiae are marked on latent fingerprints by latent print examiners, in support of the article “Interexaminer variation of minutia markup on latent fingerprints” [1]. The underlying data was collected in the “White Box” study [2]; the aspects of that data specific to interexaminer variation in minutiae markup have not been previously published.

## Experimental design, materials and methods

The test procedure, fingerprint data, and examiner determination and markup data are summarized here, and are described in greater detail in [2].

## 1 Materials and methods

### 1.1 Test procedures

Fig. 1 in [2] summarizes the test workflow, which conforms broadly to the prevailing ACE methodology. The Verification phase was not addressed. Examiners could review and revise their work prior to submitting their results. Examiners were free to modify the markup and value determination for the latent after the exemplar was presented, but any such changes were recorded and could be compared with their Analysis responses. The test procedure is described in detail in [2], including the complete test instructions and introductory video.

### 1.2 Fingerprint data

The fingerprints were collected at the FBI Laboratory and at Noblis under controlled conditions, and from operational casework datasets collected by the FBI. We provide a detailed description of the fingerprint data selection process in Appendix S.5 in [2]. All prints were impressions of distal segments of fingers, including some sides and tips.

The latents were processed using a variety of development techniques. The processed latents were captured electronically at 8-bit grayscale, uncompressed, at a resolution of 1000 pixels per inch.

The exemplars included both rolled and plain impressions captured as inked prints on paper cards or using FBI-certified livescan devices; they were captured at 8-bit grayscale, 1000 or 500 pixels per inch and either uncompressed or compressed using Wavelet Scalar Quantization [3].

The fingerprint pairs were selected to vary broadly over a four-dimensional design space: number of corresponding minutiae, image clarity, presence or absence of corresponding cores and deltas, and complexity (based on distortion, background, or processing). The primary focus was to test the boundaries of sufficiency for individualization determinations, and therefore we deliberately limited the proportion of image pairs on which we expected unanimous determinations.

We selected nonmated pairs to result in challenging comparisons either by down-selecting among exemplar prints returned by searches of the FBI’s Integrated AFIS (IAFIS) or from among neighboring fingers from the same subject.

To ensure coverage of the design space and balance of image pairs across examiners, the assignments of fingerprint images to examiners were randomized based on an incomplete block design (with examiners as blocks, image pairs as factor levels), balanced to the extent possible (using the criterion of D-Optimality).

For each image pair assigned to an examiner, the test process saved two data files: one saved upon completion of the Analysis phase (before the exemplar print was presented) and a second upon completion of the Comparison phase. The files complied with the ANSI/NIST-ITL [4] standard, using the COMP transaction described in the Latent Interoperability Transmission Specification [5].

### 1.3 Local ridge clarity

The annotations of local ridge clarity complied with the Extended Feature Set (EFS), which is part of the ANSI/NIST-ITL standard [4]. EFS defines a color-coding method for describing clarity [6]. For minutiae, the primary distinction with regard to clarity is that for green or better areas, the examiner is “certain of the location, presence, and absence of all minutiae” (White Box Instructions, Appendix 22 in [2]). Yellow areas indicate the opposite, that location, presence, and/or absence are not certain. Black or red areas should not have any marked minutia: when this occurs it is often due to imprecise painting of the clarity, or to not following instructions.^1^ For this analysis, we simplified the classification to clear (green or better) vs. unclear (yellow or worse).

Unless otherwise stated, we report the clarity as marked by that examiner. In some analyses we use the median clarity across multiple examiners, which combines the clarity maps from the examiners who were assigned that pair to represent a group consensus. This reduces the impact of outlier opinions and imprecision. When constructing the median clarity maps, we excluded four examiners whose clarity markup did not comply with the test instructions.

### 1.4 Examiner responses: determinations and markup data

As detailed in Appendix SI-5 of [2], we received valid responses from 170 participants. Each participant was assigned 22 image pairs from a pool of 320 total pairs. Early in the testing process, a problem was identified in seven image pairs; ten responses on these image pairs were excluded, yielding a total of 3730 valid responses from the Analysis phase. Examiners marked 44,941 minutiae on 3550 latents (180 Analysis-phase markups included no minutiae).

**Fig. 1:**
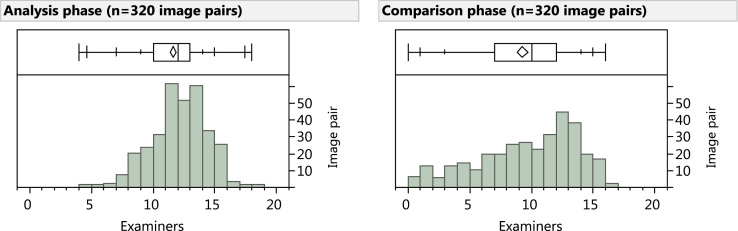
Number of valid examiner markups per image pair. (Left) Analysis phase (median 12); (Right) Comparison phase (median 10). 314 image pairs were compared by one or more examiners; 271 were compared by five or more.

Comparison-phase responses include 2966 comparisons where neither the latent nor the exemplar was assessed to be NV; this omits 2 invalid determinations (software issue) and 762 NV determinations (713 Analysis-phase latent NV, 43 Comparison-phase latent NV, and 6 Comparison-phase exemplar NV). Our previous report on changes made from Analysis to Comparison [7] omitted an additional nine responses whose Analysis-phase markup was not captured until after the exemplar had been presented. The number of valid responses per image pair is summarized in Fig. 1.

The corresponding minutia data excludes markups by five examiners who routinely did not annotate correspondences, and two markups that were missing a Comparison determination. This resulted in 3618 valid markups for analyses of corresponding minutiae (45,130 Comparison-phase minutiae marked on the latent). For some analyses, we include all minutiae marked during Analysis (including deletions) or added during Comparison (52,155 minutiae, 50,894 of which are on the 3618 markups with valid corresponding minutiae).

## 2 Example markups

**Fig. 2:**
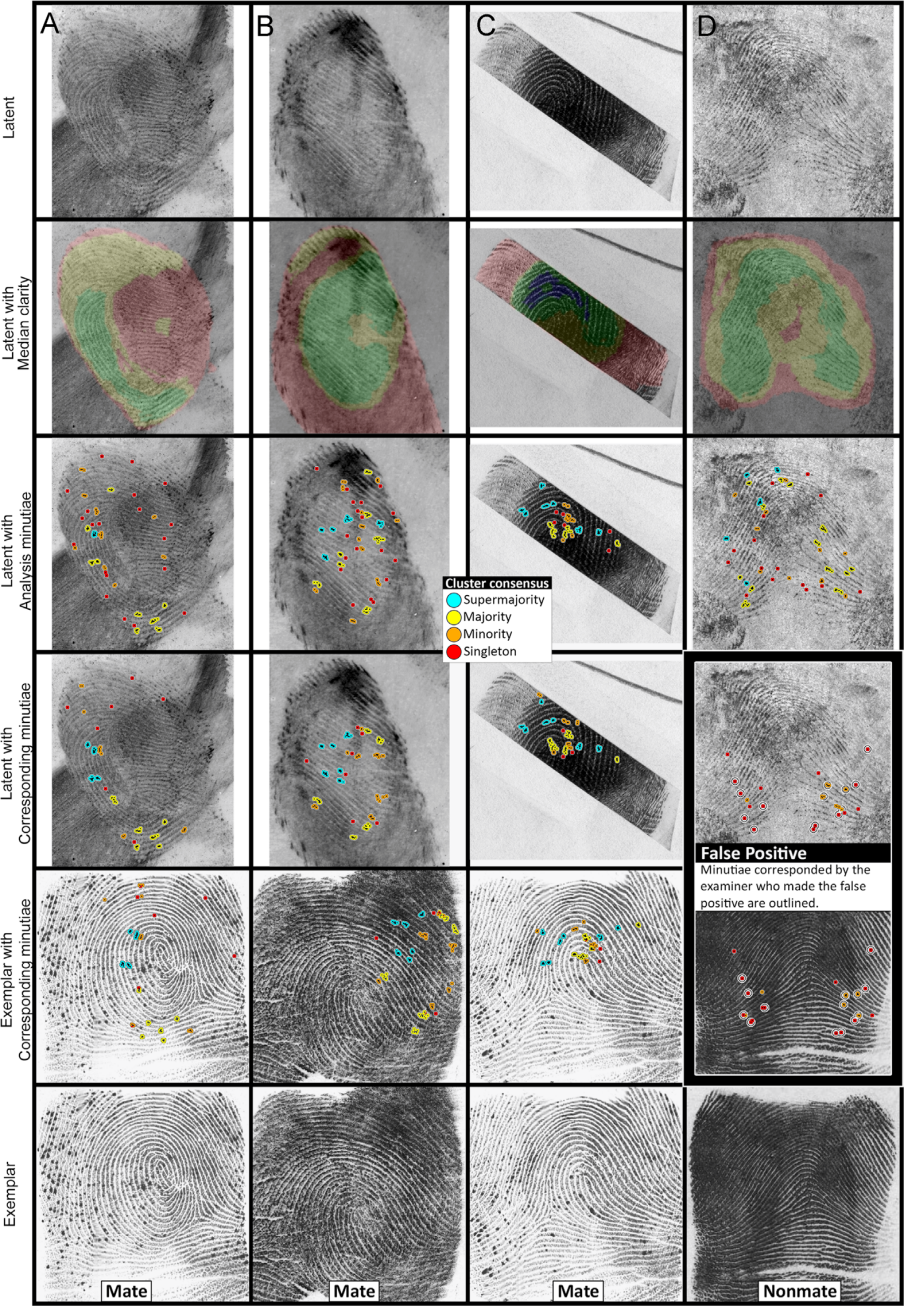
Examples of markup for four comparisons. Examiner determinations are summarized in Table 1.

Fig. 2 shows four examples of latent-exemplar pairs (columns A–D); this expands on the examples (A and B) used in Fig. 6 of [1]. Marked minutiae are shown as small black dots inside color-coded clusters. For the Analysis phase, cluster colors indicate the proportion of examiners who marked within that cluster; for the Comparison phase, colors indicate the proportion of comparing examiners who corresponded the minutia as marked on the latent. The third row of images ("Latent with Analysis minutiae") shows all minutiae as marked in the Analysis phase; the fourth row ("Latent with corresponding minutiae") shows markup from the Comparison phase limited to those minutiae that examiners marked as corresponding; the fifth row ("Exemplar with corresponding minutiae") shows the locations of the corresponding minutiae as marked on the exemplar. Because marked minutiae from one cluster on the latent did not always correspond to one cluster on the exemplar (either due to examiner disagreements or behavior of the clustering algorithm), the fifth row ("Exemplar with corresponding minutiae") uses the color-coding from the latent markup to help visualize the correspondences.

Table 1 describes for each of the four examples shown in Fig. 2, the number of examiners contributing to the clusters, and their determinations.

Note that example D is the one comparison on which an erroneous individualization occurred (also shown as an example in Fig. 2 of [7]). Five examiners marked correspondences (two of whom also marked discrepancies), one additional examiner marked debatable correspondences, and one additional examiner marked discrepancies. Even after omitting the examiner who individualized, more correspondences were marked on this image pair (22, in 11 clusters) than on any other nonmated image pair in the test. Other top examples of nonmated image pairs with many correspondences marked included one with 18 correspondences (in 12 clusters, by two of ten comparing examiners), and another with 13 correspondences (in 8 clusters, by five of eight comparing examiners).

## 3 Effect of clustering parameters

Examiners’ markups differed in whether or not individual minutiae were marked, and in the precise location where the minutiae were marked. In order to focus on whether examiners agree on the presence or absence of minutiae, we need to see past minor variations in minutia location. Neumann et al. [8] used ellipses to determine whether two minutiae should be considered the same, based on an expectation of more variation in location along the direction of the ridge than perpendicular to ridge flow; here we did not collect minutia direction, making this approach impractical. In [7], our technique of classifying features as retained, moved, added or deleted was based on a fixed radius of 0.5 mm (0.02 in., or approximately the average inter-ridge distance) — although that approach was satisfactory for two markups where one was derived from the other, it is not well suited to comparing more than two markups.

We used automated clustering algorithms in order to classify minutiae marked by multiple examiners as representing the same minutia on the latent. Clustering was implemented in two stages as follows:

1. For each fingerprint, the set of all minutiae *x*,*y* coordinates (as marked by the examiners) was preliminarily clustered using DBSCAN with a given radius *r*, and no lower limit to the cluster size. That is, singletons were treated as valid clusters, not labeled as “noise.”
2. Oversized preliminary clusters were split using agglomerative hierarchical clustering, with ceiling (mean number of marks per examiner) as the cutoff point. Hierarchical clustering assembles a tree of cluster relationships; there is no assumption of a fixed radius.

**Fig. 3:**
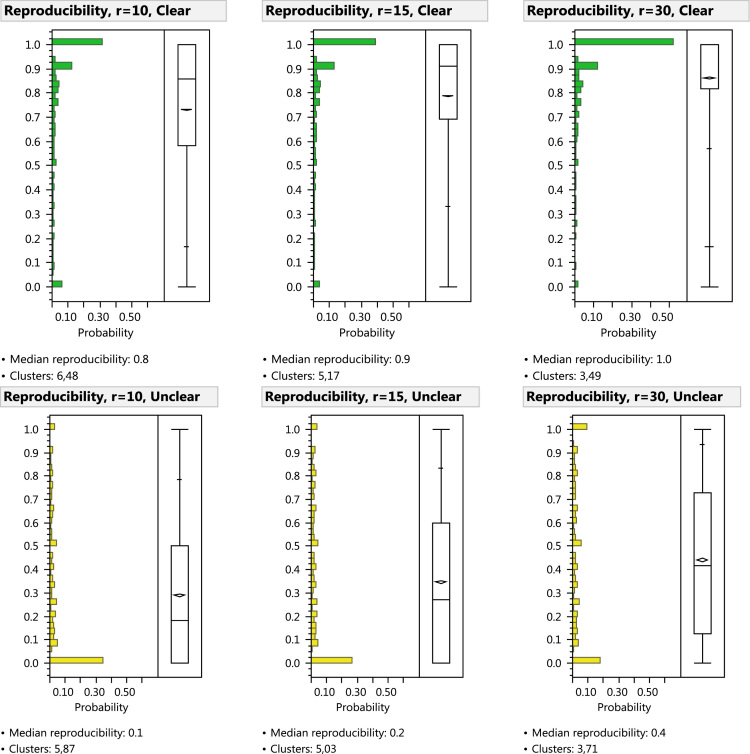
Histograms showing effects of varying DBSCAN reachability distance (*r*=0.010", 0.015", 0.030") on reproducibility measure. Comparison-phase minutia reproducibility distributions after DBSCAN clustering: oversized clusters were not split.

Neither algorithm makes use of any information from the fingerprint images themselves; they rely entirely on the *x*,*y* coordinates of the minutiae as marked by examiners. The implementation of Density-based Spatial Clustering of Applications with Noise (DBSCAN) we used was written by Michal Daszkyowski of the University of Silesia in 2004. [9], [10] 1 The DBSCAN radius was set to 0.015" (0.38 mm) after extensively reviewing the algorithm׳s performance over a range of radius settings. In our review, we considered several standard clustering performance measures and visually assessed the resulting clusters as plotted superimposed over the latent prints. As shown in Fig. 3 and Table 2, any choice of radius substantially biases the reproducibility distributions: increasing the radius increases the measured mean reproducibility, and decreases the measured number of clusters. We selected a slightly large radius in order to aggregate some of the less precisely focused clusters; we then split many of the oversized clusters in the second step.

Oversized preliminary clusters were selected for subsequent splitting by agglomerative hierarchical clustering based on a criterion of (mean number of marked minutiae per examiner) >1.5. This arbitrary threshold was selected because (1) automated splitting of clusters meeting this criterion was highly successful, and (2) for lower values (between 1 and 1.5), it was usually not apparent even to a human how to split correctly without careful interpretation of the fingerprint image. The oversized preliminary clusters often contained multiple, clearly distinct ridge events, but otherwise were difficult to resolve by visual inspection. We used MATLAB׳s implementation of agglomerative hierarchical clustering algorithm; Ward׳s method was selected for computing the distance between clusters.^2^ Ward׳s method helps overcome the main flaw of DBSCAN, which is that it tends to fail when faced with highly heteroskedastic data (data in which the variance differs among subsets).

Clustering was performed separately on Analysis markup (*n*=44,941 minutiae), Comparison markup (*n*=46,205 minutiae), and combined markup (*n*=52,155 minutiae). Combined markup (used in sections 9 and 10.2) includes both deleted and added minutiae. 94% of the Analysis-phase clusters have a maximum radius less than 1 mm; 99.2% less than 1.5 mm; 99.95% less than 2 mm.

## 4 Minutia reproducibility and consensus (Analysis phase)

### 4.1 Reproducibility and consensus by clarity

**Fig. 4:**
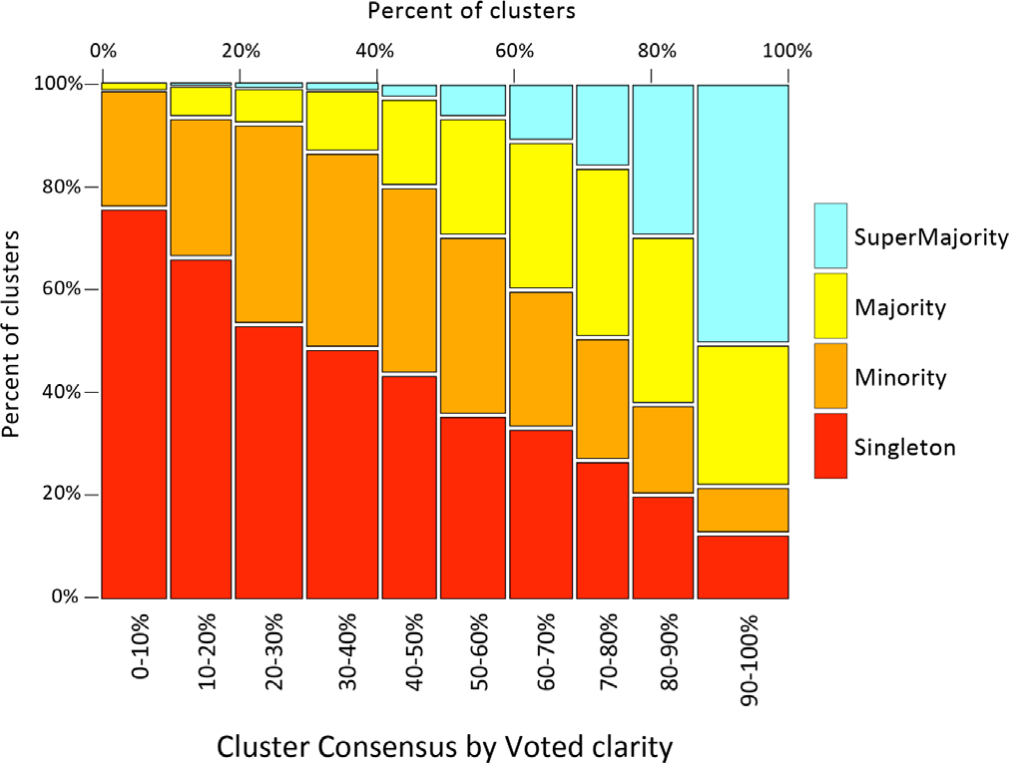
Consensus by voted clarity (Analysis phase, *n*=10,324 clusters). Compare to Fig. 9 in [1], which shows reproducibility by voted clarity.

**Fig. 5:**
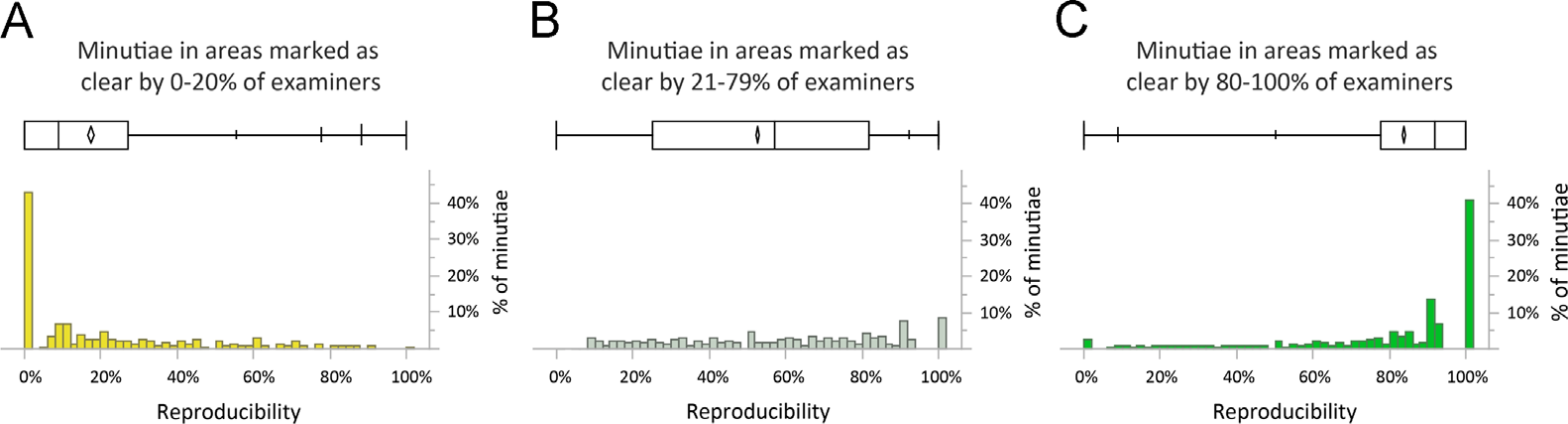
Reproducibility by voted clarity in areas (A) that examiners agree are unclear; (B) where examiners do not agree on clarity; (C) that examiners agree are clear. (Analysis phase, *n*=44,941 minutiae). Mean reproducibility=(A) 17%; (B) 53%; (C) 84%.

Table 3, Table 4 and Fig. 4, Fig. 5 describe associations between reproducibility and clarity, and between consensus and clarity. While clarity as painted by the examiners who marked the minutiae is a strong predictor of reproducibility, consensus descriptions of clarity provide a better explanation of interexaminer variation in minutiae markup.

Minutiae that were more highly reproduced were more likely to be found in clear areas of the latent. Table 4 illustrates how median clarity explains this association better than examiner clarity.

The latent prints included many areas where examiners did not agree on clarity. Fig. 4 indicates how these areas of “debatable clarity” contribute to reproducibility, by showing the associations between consensus and clarity.

Fig. 5 shows the distribution of minutia clarity conditioned on the proportion of examiners describing that location as clear: minutia reproducibility is very high when examiners concur that a location is clear, very low when examiners concur that a location is unclear, and varied when there is no concurrence on clarity. This can explain some of the lack of association seen in Fig. 4.

### 4.2 Reproducibility of entire markups

In addition to assessing interexaminer variability by minutiae (reproducibility) and by clusters (consensus), we can assess variability by entire markups. Table 5 describes the extent to which the examiners’ minutia markup was in complete (or near-complete) agreement on each latent, conditioned on the presence of clear minutiae and majority clusters.

### 4.3 Singletons and solo misses

Table 6 shows the distribution of singletons per markup. With a mean of 12 examiners per latent, 50% of the Analysis-phase markups had singletons. 15% of all markups had more than two singletons, and these markups accounted for 59% of all singletons. 6.6% of examiner clear minutiae were singletons; 16.8% of examiner unclear minutiae were singletons.

Analogous to singletons are “solo misses,” i.e., minutiae that were marked by all but one of the examiners. Unlike singletons, solo misses occur primarily in clear areas: there were a total of 640 solo misses during Analysis (6% of clusters), 610 of which were in median clear areas. Although singletons are far more numerous than solo misses, solo misses disproportionately affect measures such as mean reproducibility, because reproducibility counts each singleton once (as reproducibility=0) while it counts solo misses once for each examiner who marked that minutia (e.g., as mean reproducibility=92% if 11 of 12 examiners marked a minutia).

### 4.4 Reproducibility of minutia with respect to value determinations

**Fig. 6:**
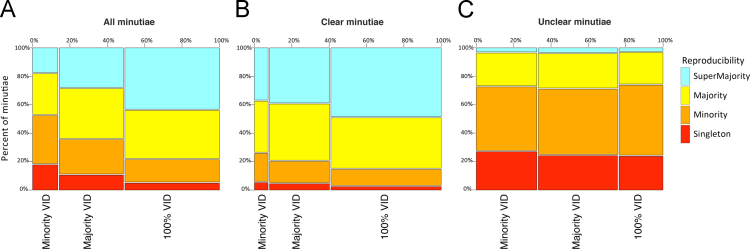
Association between latent value determinations and reproducibility. (A) all minutiae (Analysis phase, *n*=44,941 minutiae); (B) median clear minutiae (*n*=33,846 minutiae); (C) median unclear minutiae (*n*=11,095 minutiae).

Minutia reproducibility tended to be higher on latents that examiners agreed are VID than those that examiners agreed are not VID. However, as shown in Fig. 6, most of this association can be accounted for in terms of differences in clarity: those latents that examiners agreed are VID tend to have more minutiae marked in clear areas.

We have previously reported [2], [7] that when one examiner assesses a latent to be VID and another examiner assesses that same latent to be NV, the examiner assessing the latent to be VID can be expected to mark more minutiae. Here we take a closer look at how differences in value assessments relate to whether examiners mark specific minutiae.

The following logistic regression model was used to estimate the probability that an examiner would mark a minutia given the level of consensus for that minutia and the examiner׳s value assessment. This model allows us to estimate how much effect is specifically associated with the value assessments as opposed to other factors such as clarity or which regions of the prints examiners chose to mark that are largely accounted for by conditioning on consensus:

(1) $$\mathrm{logit}(\pi )=\beta_{0}+\beta_{\mathrm{Value}}*\mathrm{Value}+\beta_{\mathrm{Consensus}}*\mathrm{Consensus},$$

where π is the probability that this examiner marked the minutia given this examiner׳s value assessment of the latent and given the proportion of all examiners who marked this minutia. The probability estimates are summarized in Table 7. Even after accounting for the level of consensus on each minutia, examiners are more likely to mark minutiae when they assess a latent to be VID.

**Fig. 7:**
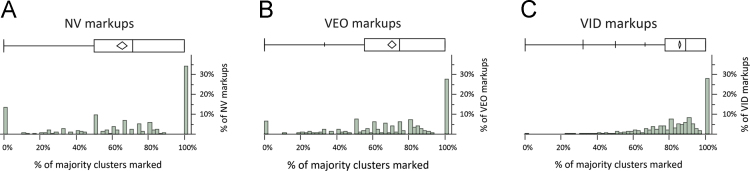
Percentage of majority clusters marked, conditioned on value assessment (Analysis phase, *n*=3588 markups=(A) 602 NV+(B) 570 VEO+(C) 2416 VID; 142 of the 3730 markups had no majority clusters).

**Fig. 8:**
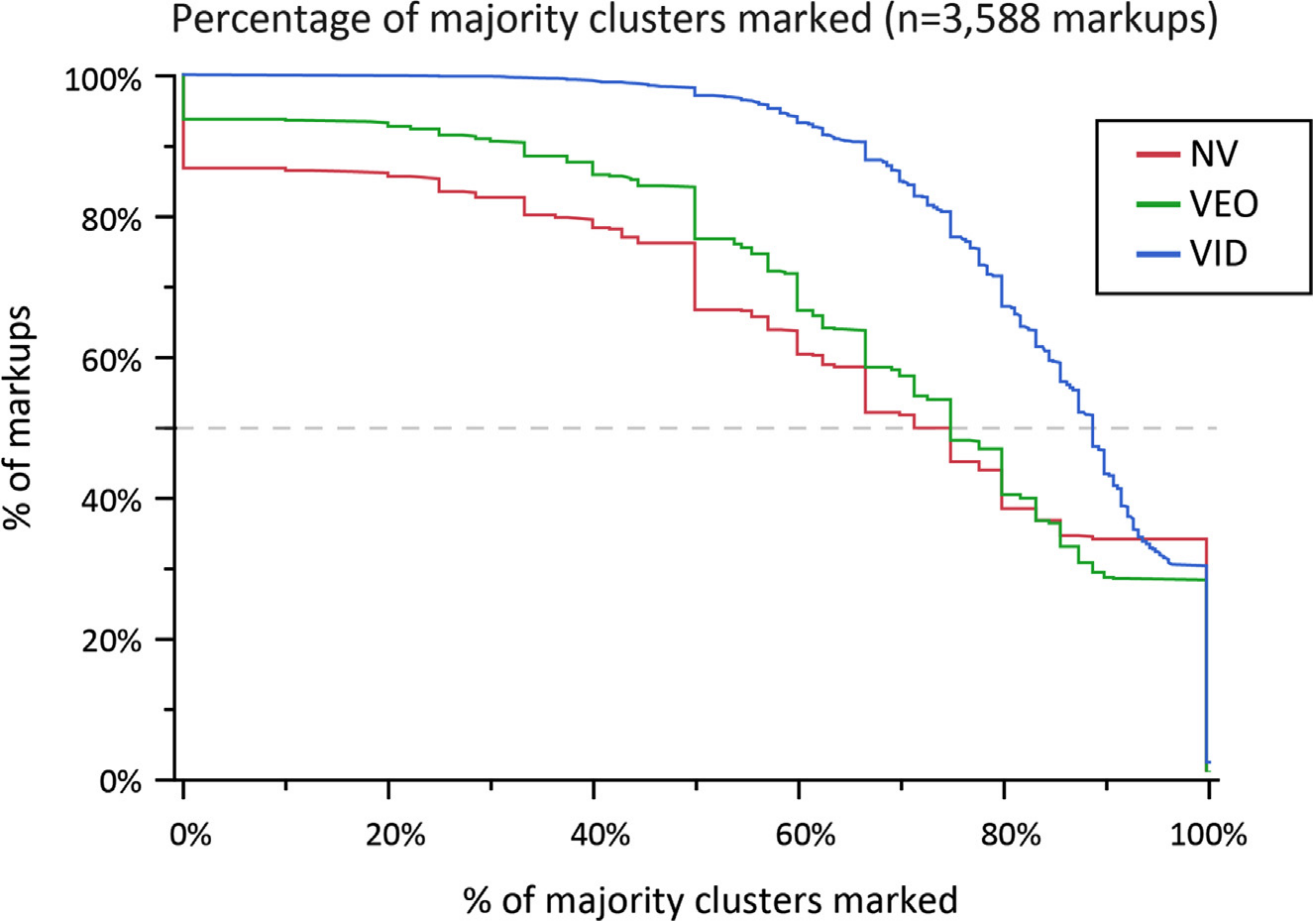
Cumulative distribution functions of the percentage of majority clusters marked, conditioned on value assessment (same data as Fig. 7). The median number of majority clusters marked (dashed line) was 71% of NVs; 75% of VEOs; 89% of VIDs. No majority clusters were marked (left extreme) on 13% NV latents; 6% of VEO latents; and 0% of VID latents. All majority clusters were marked (right extreme) on 34% NVs; 27% VEOs; and 28% VIDs.

The decisions to mark or not mark minutiae on a single latent are not independent events. For example, examiners occasionally mark no minutiae on latents assessed to be NV or VEO; this may contribute to the lower probability of examiners marking minutiae in majority clusters on these responses. Taking this lack of independence into account, we realize that conditioning on the level of consensus, as shown in Table 7, does not completely remove the confounding effects of factors such as clarity. Fig. 7, Fig. 8 show that when examiners assessed latents to be VID, they almost always marked most of the majority clusters; when they assessed latents to be NV or VEO, they often marked fewer than half of the majority clusters.

Table 8, Table 9 summarize Analysis-phase reproducibility by latent value assessment and clarity.

## 5 Reproducibility of nonminutia features

**Fig. 9:**
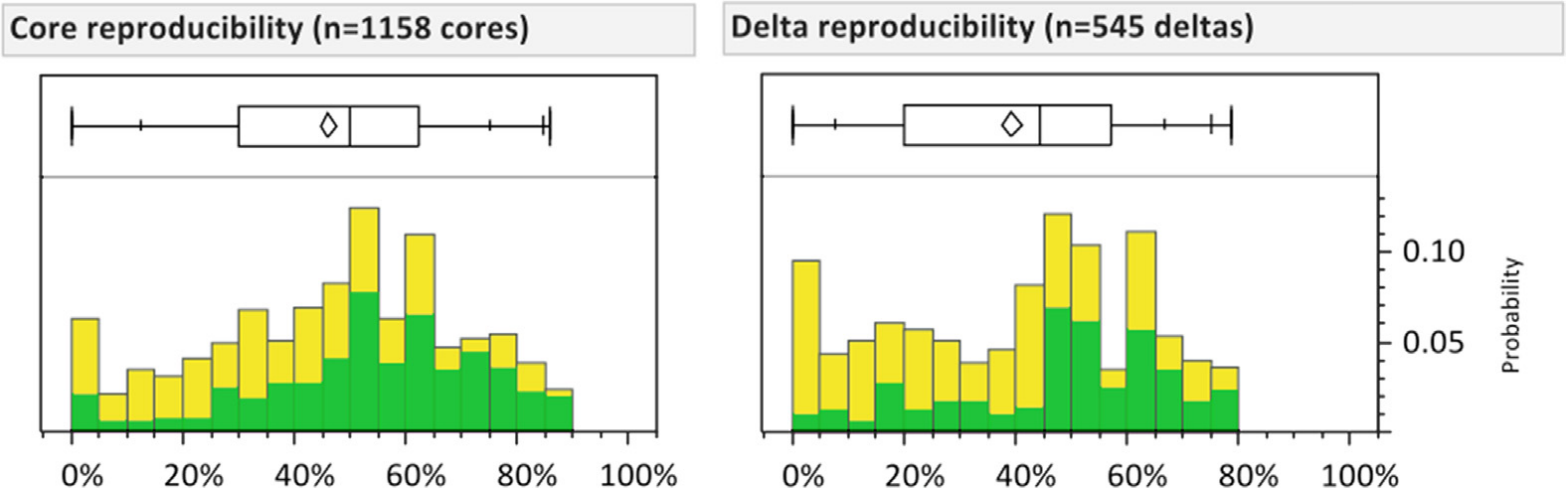
Reproducibility of cores and deltas, Analysis phase. Here we gauge reproducibility based on a 1.5 mm (0.06") radius (corresponding to our instructions that cores and deltas could be located within approximately three ridge intervals). Data is color-coded by examiner clarity: green (dark shading)=clear, yellow (light shading)=unclear. (Figure is reproduced in color in the web version of this article.)

Fig. 9 shows reproducibility of cores and deltas. Examiners were instructed to mark all cores and deltas on the latents, provided they could be located within approximately three ridge intervals. On those latents that had one or more cores or deltas marked by any examiners, typically only about half of the examiners marked them: no cores or deltas were unanimously marked.

Table 10 shows the prevalence of nonminutia features in the area of minutia clusters. Features other than minutiae were sometimes present in or near minutia clusters, which could indicate a disagreement as to whether a feature should be marked as a minutia, a nonminutia feature, or both. However, this did not explain much of the interexaminer variability: only 4.5% of clusters contained features other than minutiae.

## 6 Agreement in clarity markup (Analysis phase)

Examiners often disagreed as to whether or not minutiae were present and as to whether the locations of minutiae were sufficiently clear to be certain of the presence or absence of minutiae.

**Fig. 10:**
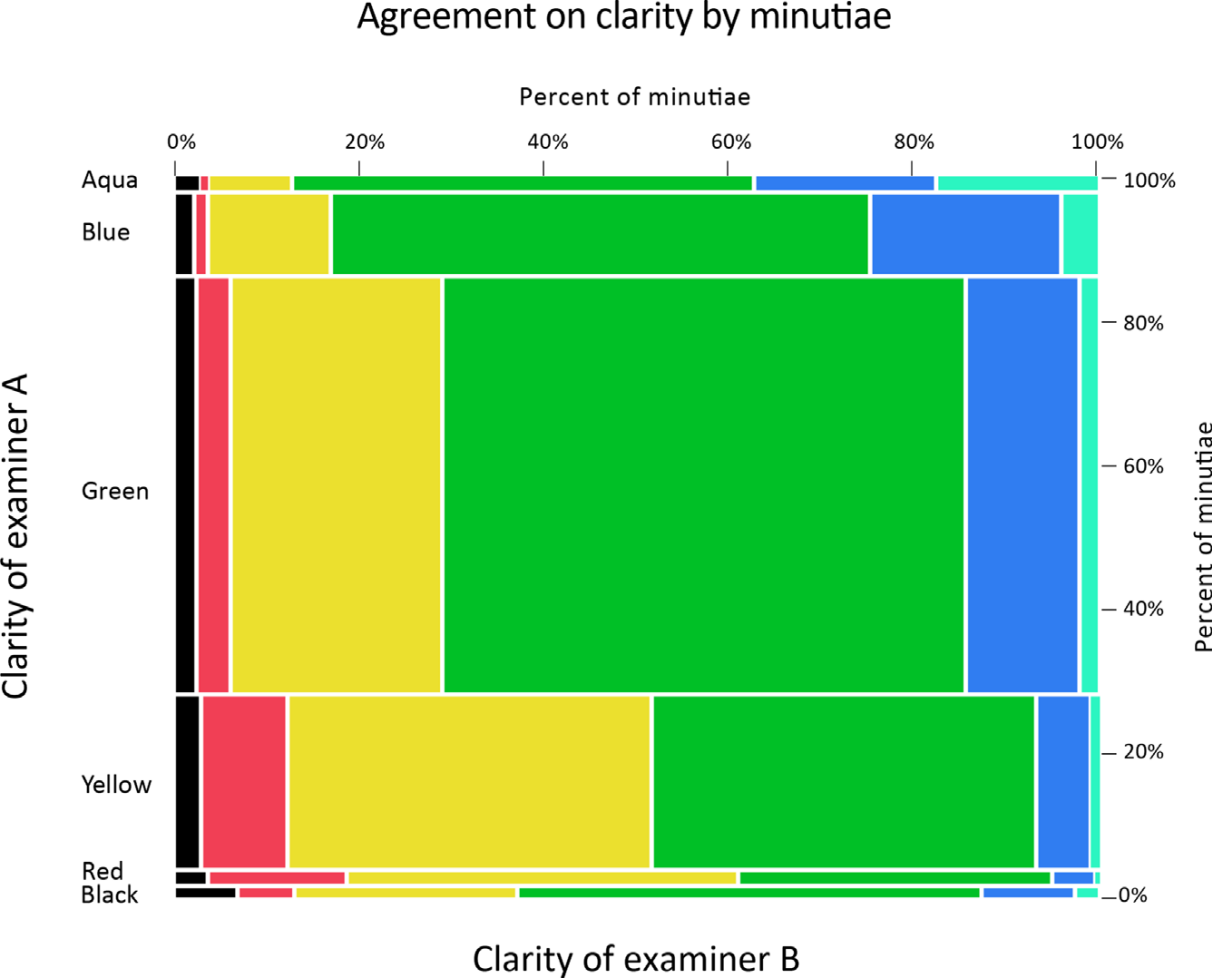
Examiner B clarity by examiner A clarity for each ***minutia*** marked by examiner A. Same data as Table 11, shown graphically, color-coded by examiner B clarity.

Table 11 and Fig. 10 show for every minutia (*n*=44,941) the distribution of clarity assigned to that location by other examiners, regardless of whether the other examiners marked a minutia at that location. When an examiner marked a minutia in an area that that examiner described as unclear, other examiners were about equally likely to describe that area as clear or unclear.

**Fig. 11:**
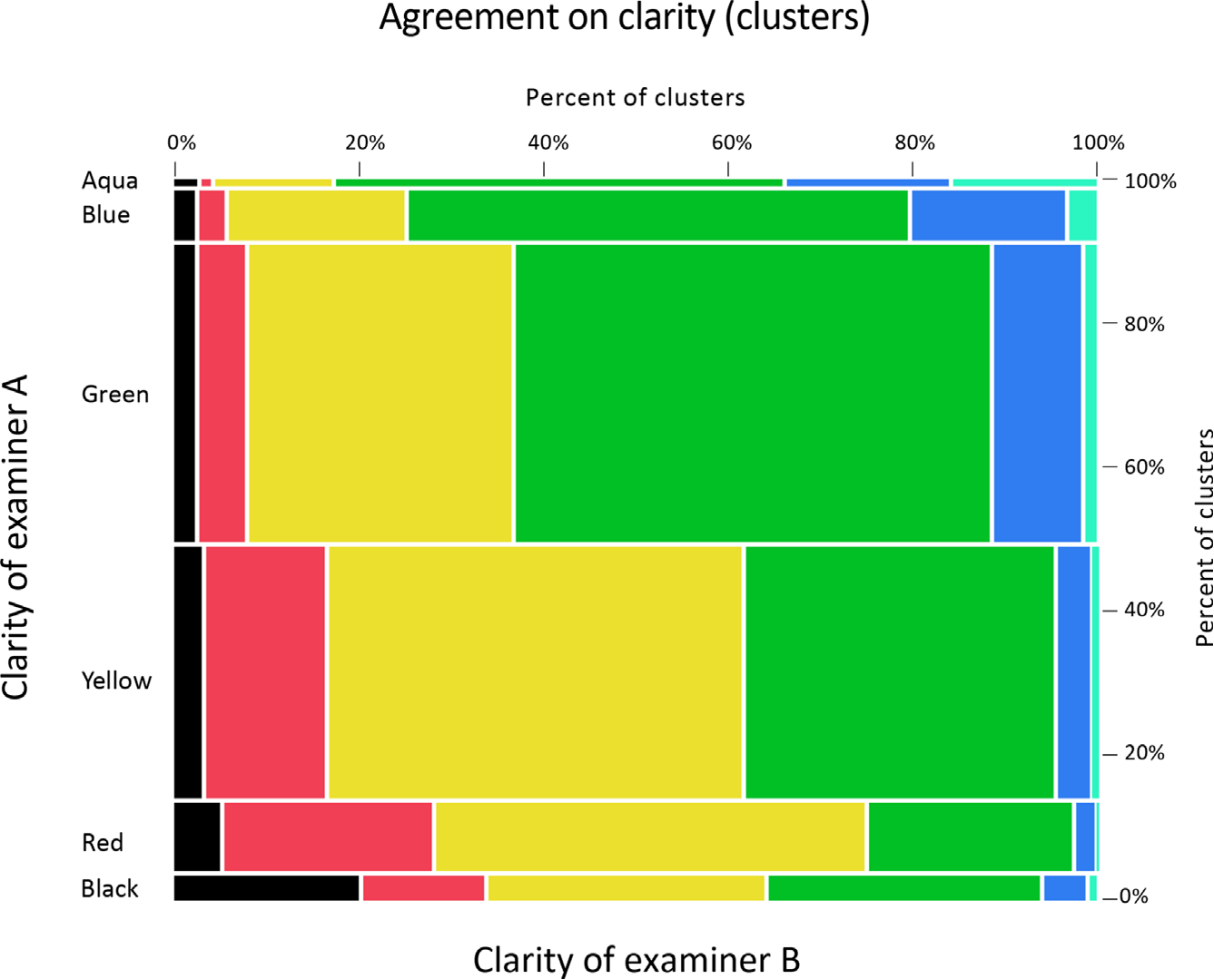
Examiner B clarity by examiner A clarity at each ***cluster*** center. Same data as Table 12, shown graphically, color-coded by examiner B clarity.

Table 12 and Fig. 11 show for every cluster center (n=10,324) the distribution of clarity assigned to that location by pairs of examiners, regardless of whether those examiners marked a minutia at that location. Selecting examiner pairs and cluster centers at random, the probability of the two examiners agreeing whether to describe that location as clear vs. unclear was 65%.

Table 13 shows for every minutia marked (*n*=44,941) the distribution of clarity assigned to that location by other examiners, conditioned by whether the second examiner marked at that location. When a second examiner agreed on the presence of a minutia, that examiner was much more likely to describe the location as clear, whereas if the second examiner did not mark the minutia, that examiner was likely to describe the location as unclear.

## 7 Differences in regions with marked minutiae

**Fig. 12:**
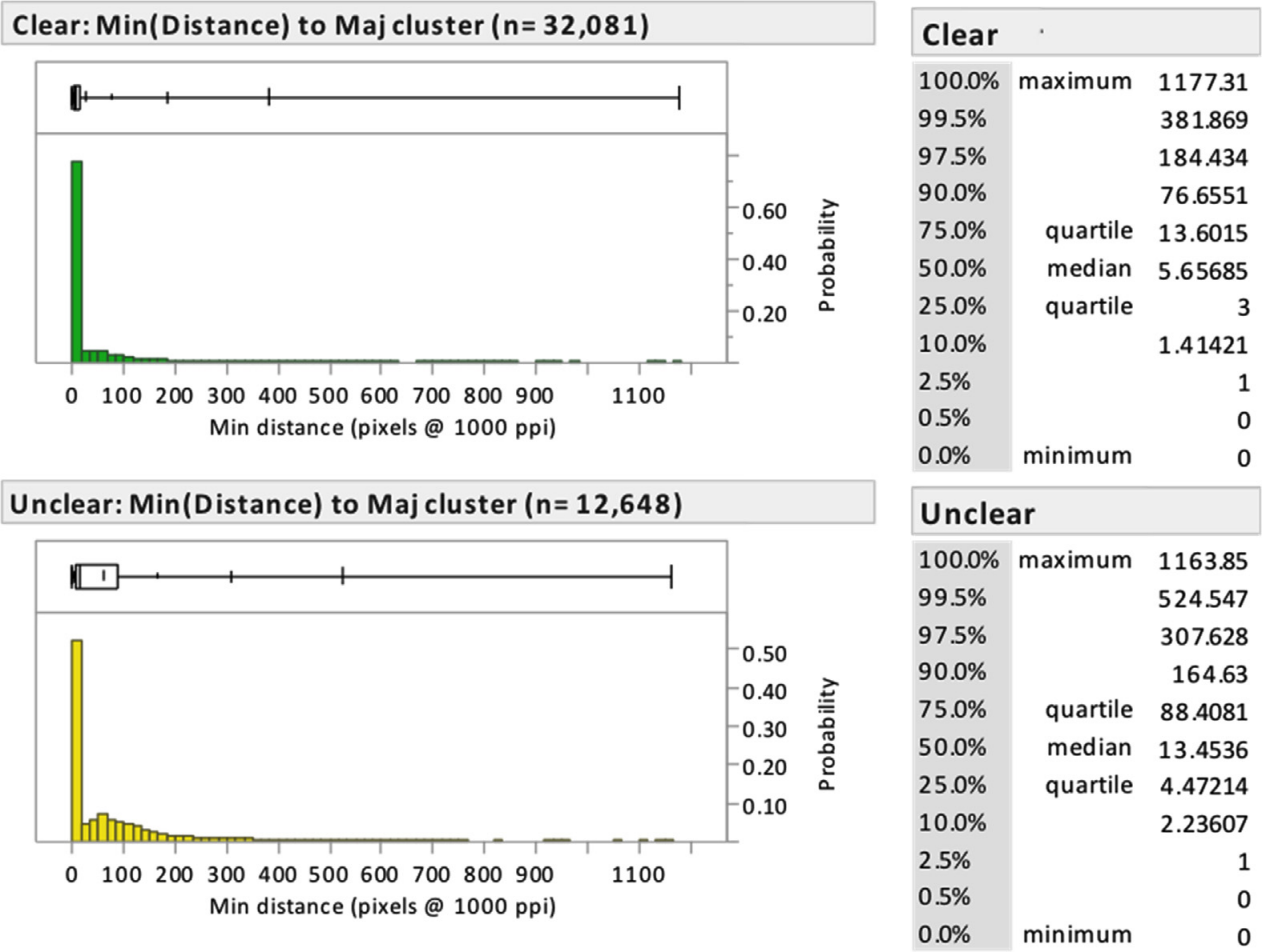
Distance of *Analysis-phase* minutiae to nearest majority cluster by examiner clarity. Distance is measured in units of 0.001”. (Analysis phase, *n*=44,729; another 212 minutiae were marked on latents having no majority clusters).

**Fig. 13:**
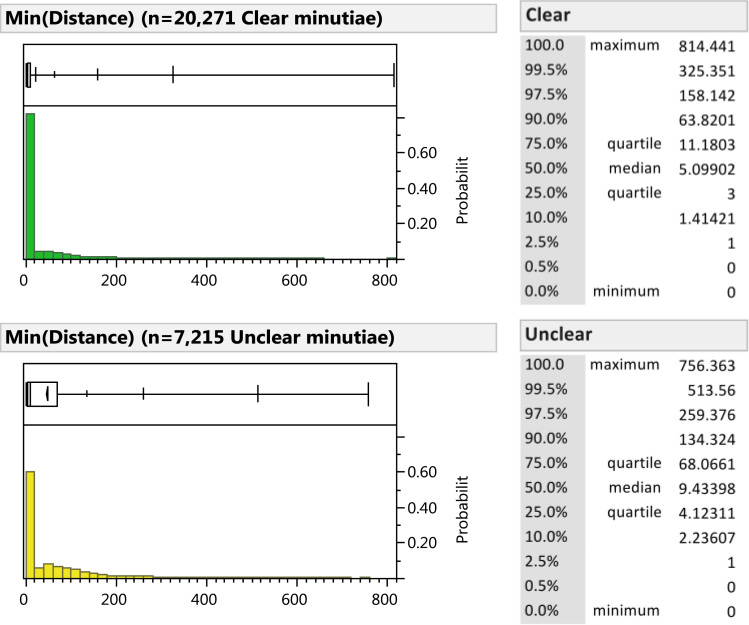
Distance of *corresponding* minutiae to the nearest cluster corresponded by a majority of comparing examiners, by examiner latent clarity. Distance is measured in units of 0.001”. The set of majority clusters was limited to those in which at least three examiners marked corresponding minutiae; "majority" was calculated among those examiners who marked at least one correspondence on the image pair. (Comparison phase, *n*=27,486; another 454 corresponding minutiae were marked on latents having no majority cluster).

Some examiners mark minutiae far away from those marked by other examiners. This may be due to disagreements regarding the boundaries of the impression being considered (i.e., the region of interest), or disagreements on which areas in the region of interest are of sufficient quality to mark minutiae. Table 14 describes what proportion of minutiae were marked far from the nearest majority cluster. Fig. 12 (Analysis phase) and Fig. 13 (corresponding minutiae, Comparison phase) show the distributions of the distances from marked minutiae to the nearest majority cluster.

## 8 Consensus and sufficiency (Analysis and comparison phases)

Previously, we reported [2] that the number of minutiae annotated by examiners is strongly associated with their own value and comparison determinations, and that seven minutiae was an approximate “tipping point”: “for any minutia count greater than seven, the majority of value determinations were VID, and for any corresponding minutia count greater than seven, the majority of comparison determinations were individualization.” Across multiple examiners, a *mean* of seven corresponding minutiae was also the point at which approximately 50% of examiners individualized (approximately 50% of examiners assessed latents to be VID when the mean minutia count was seven).

**Fig. 14:**
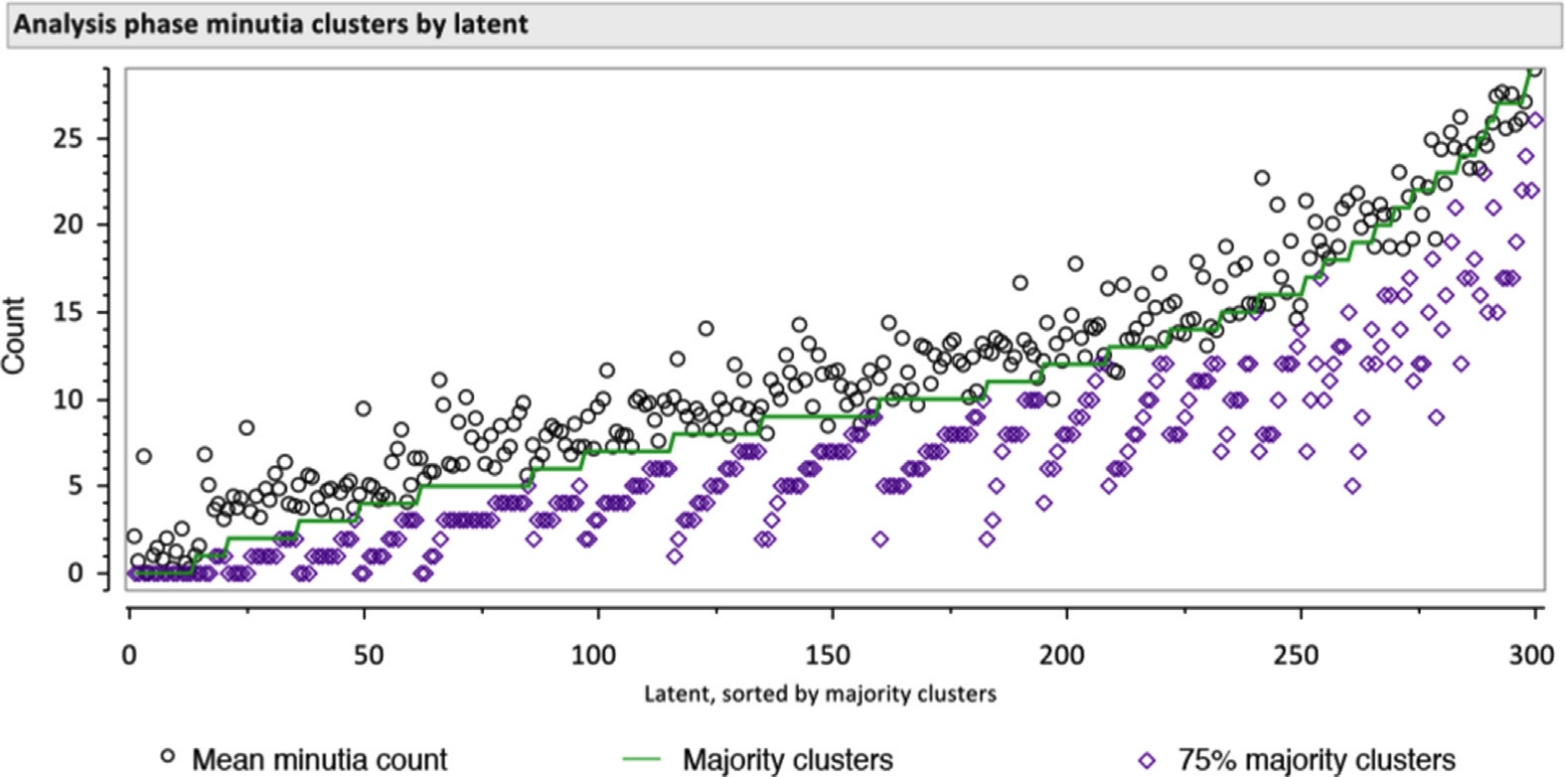
Relation among mean minutia counts and majority clusters (Analysis phase, *n*=301 latents). Latents (*x*-axis) are sorted by the number of majority clusters. Shows the mean minutia count (black circles), number of majority clusters (green line), and number of clusters marked by at least 75% of examiners (purple diamonds). (Figure is reproduced in color in the web version of this article.)

Here we report similar thresholds as measured by consensus on minutia clusters. We find counts of majority clusters comparable to mean minutia counts as predictors of examiner determinations. For example, when predicting VID determinations using logistic regression, *r*^2^=0.4253 for mean minutia counts vs. *r*^2^=0.4310 for majority clusters. As shown in Fig. 14, these majority cluster statistics are highly correlated with the mean number of minutiae, which tends to be slightly larger than the number of majority clusters.

**Fig. 15:**
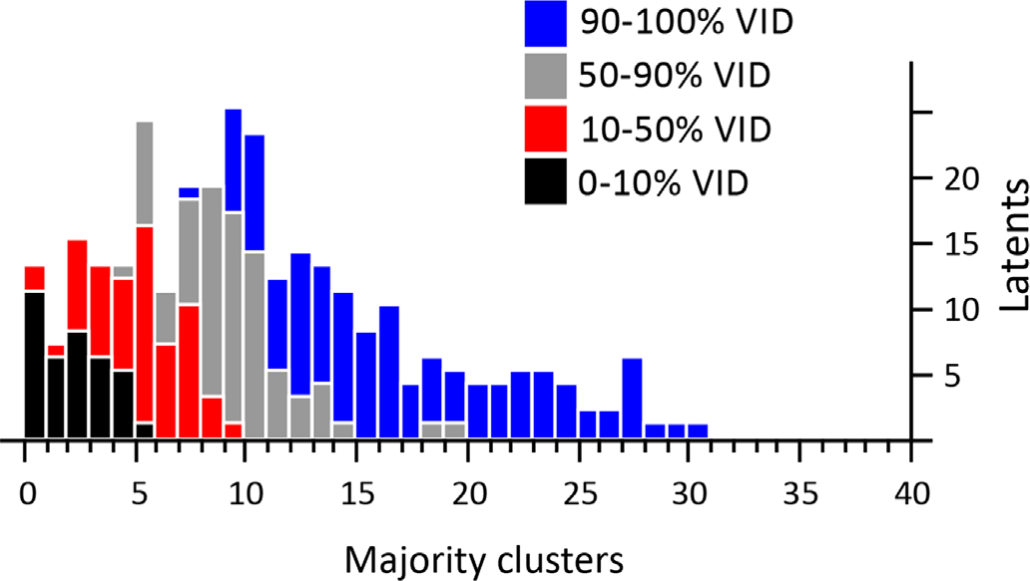
Distribution of the number of majority clusters in latents, shaded to indicate percentages of examiners who assessed each latent as VID (*n*=301 latents). Overall distribution reflects data selection for the test.

**Fig. 16:**
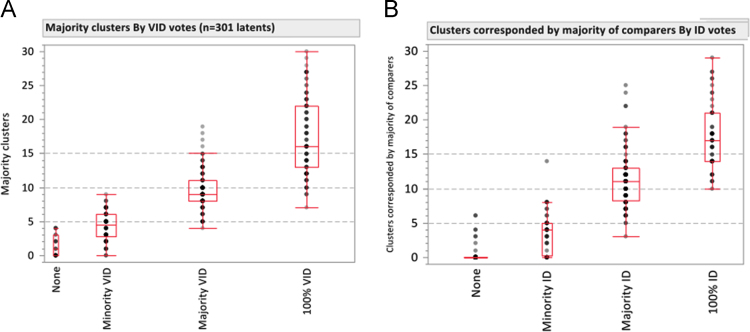
Majority minutia clusters by proportion of examiners determining (A) value for individualization (*n*=301 latents), (B) individualization (*n*=271 image pairs). *Y*-axis in chart B is the number of clusters corresponded by a majority of comparers: (number of corresponding examiners/number of comparing examiners) ≥0.5. Data excludes image pairs with fewer than five Comparison markups. One data point at *y*=65 (100% ID) not shown in (A). One data point at *y*=42 (100% ID) not shown in (B).

As shown in Fig. 15, Fig. 16A, latents with fewer than 5 majority clusters were usually not assessed as VID; latents with 10 or more majority clusters were usually assessed to be VID. Fig. 16B shows a similar association for clusters corresponded by the majority of comparing examiners: almost all image pairs with 7 or more clusters that were corresponded by a majority of comparing examiners were individualized by the majority of examiners; almost no image pairs with 5 or fewer majority corresponding clusters were individualized by the majority of examiners.

**Fig. 17:**
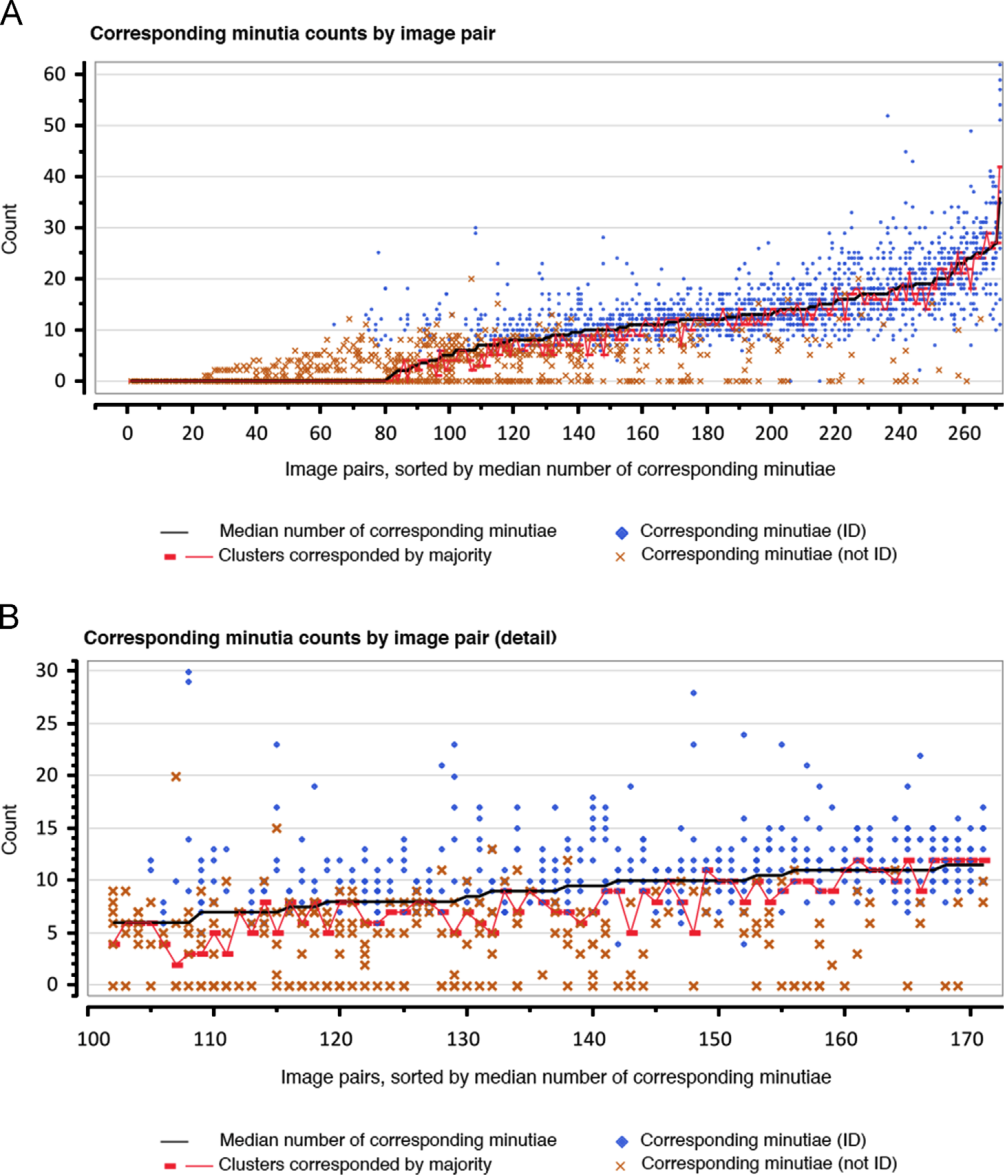
Corresponding minutiae counts by image pair: median corresponding minutiae (black line); clusters corresponded by a majority of comparing examiners (red rectangle); counts by examiners who individualized (blue diamond); counts by examiners who did not individualize (orange x). (A) 271 image pairs compared by at least 5 examiners; (B) a subset of the data enlarged to reveal interexaminer variability on 70 image pairs having 6–10 median corresponding minutiae.

In [2] we included several figures to show the association between minutia counts and value determinations, and between corresponding minutia counts and comparison determinations. Fig. 17 is comparable to Fig. 5 of [2] except that it includes a data series for the number of clusters corresponded by a majority of examiners who compared the image pair; it also includes data for both mated and nonmated image pairs. In general, the number of majority clusters tends to be approximately equal to the mean minutia count.

## 9 Reproducibility of analysis-comparison changes

**Fig. 18:**
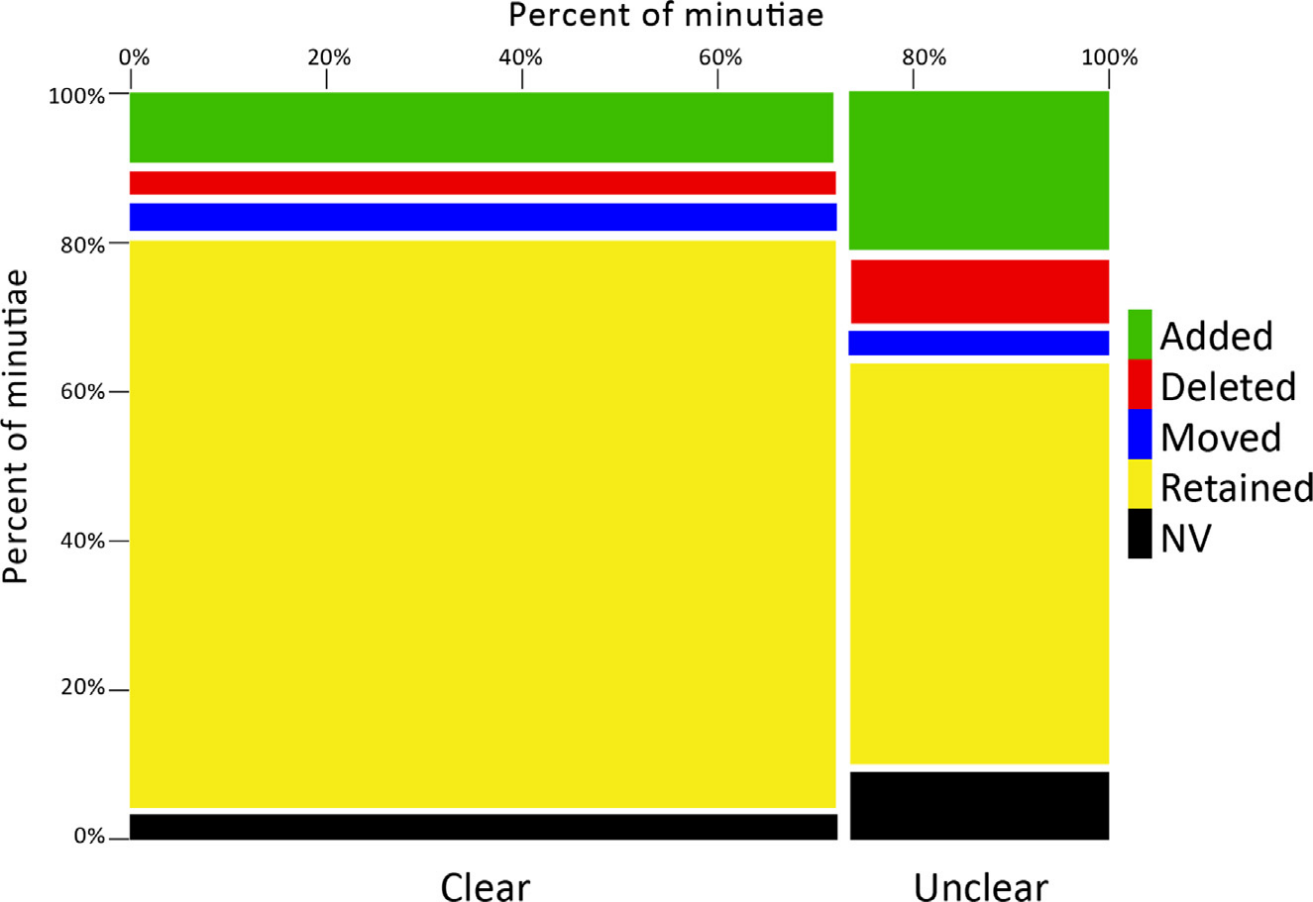
Analysis-Comparison changes by examiner clarity. Chart represents all 52,155 minutiae marked during either the Analysis or Comparison phases.

As previously reported, examiners often modified their latent Analysis markup during the Comparison phase [7]. For each pair of latent markups (analysis and comparison phases), we classified features as retained, moved, deleted, or added. A retained feature is one that is present at exactly the same pixel location in both markups; a moved feature refers to one that was deleted during Comparison and replaced by another within 0.5 mm (approximately one ridge width); a deleted feature is one that was present in the Analysis markup only (no Comparison feature within 0.5 mm); an added feature is one that was present in the Comparison markup only (no Analysis feature within 0.5 mm). Fig. 18 summarizes the extent of such changes, by clarity, showing that unclear minutiae were much more likely to be changed.

Table 15, Table 16 show that deleted and added minutiae are strongly associated with low reproducibility. This association is stronger in clear areas than unclear areas: using logistic regression to predict deletions and additions from minutia reproducibility, we find that for deleted minutiae, *r*^2^=0.1243 (clear) and 0.0686 (unclear); for added minutiae, r^2^=0.0640 (clear) and 0.0332 (unclear).

Having shown that reproducibility and clarity are strongly associated, we took a closer look at how reproducibility and clarity are associated with changes. We used logistic regression to model deleted and added minutiae as responses to reproducibility and clarity. Predicting deleted minutiae from reproducibility and examiner clarity (*r*^2^=0.1114), only the reproducibility term is significant; clarity provides no additional information (using median clarity makes no meaningful improvement to the model: *r*^2^=0.1116). Predicting added minutiae from reproducibility and examiner clarity (*r*^2^=0.0762), both terms are significant, though the reproducibility term contributes much more than clarity (predicting added minutiae from reproducibility alone results in *r*^2^=0.0682; from examiner clarity alone, *r*^2^=0.0271; from median clarity alone, *r*^2^=0.0359). Examiners are more likely to add minutiae in low-clarity areas even after accounting for reproducibility of those minutiae. Our ability to predict deleted minutiae is not further improved by knowing clarity after accounting for reproducibility.

**Fig. 19:**
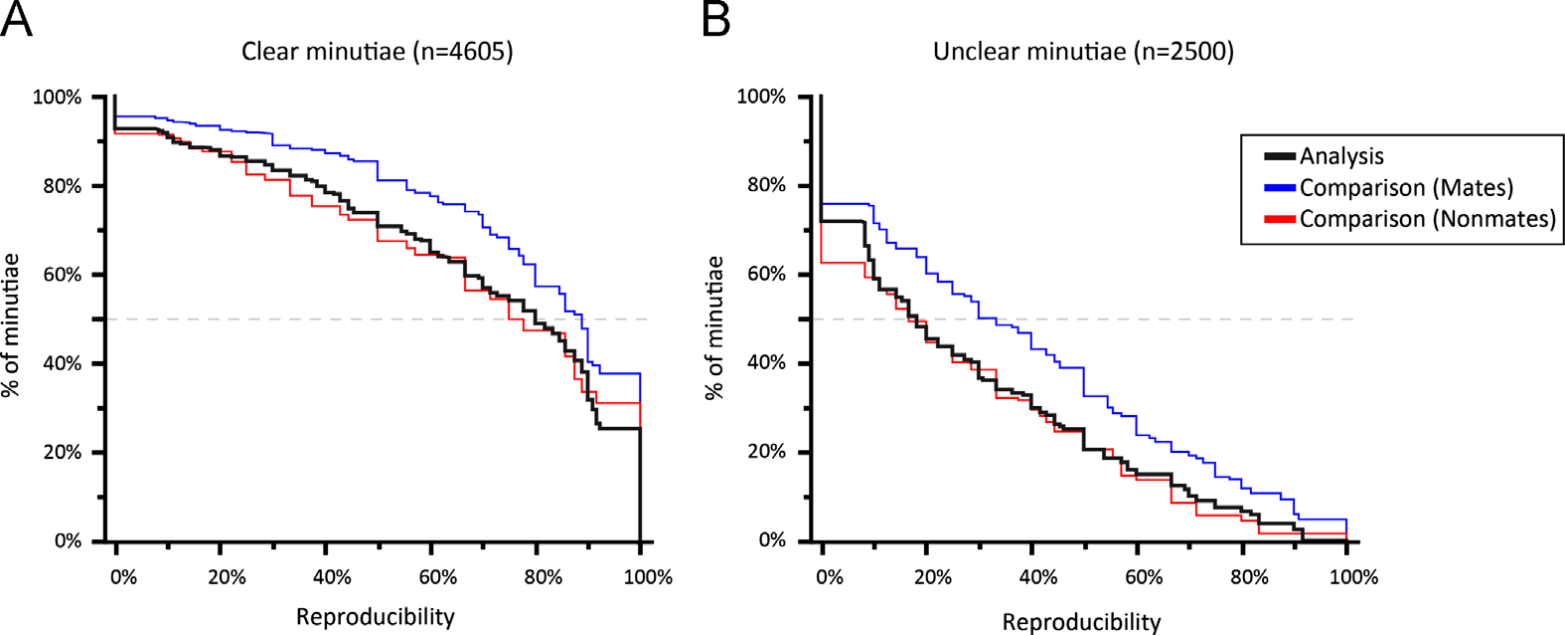
Minutia reproducibility in Analysis to Comparison phases, by median clarity. Y-axis indicates the percentage of minutiae that meet or exceed the *x*-axis reproducibility level. Data is limited to 19 latents that were presented to examiners in both mated and nonmated pairings: 302 markups (179 mated, 173 nonmated) where the examiner proceeded to Comparison (latent was not assessed NV). On the mated pairs, median reproducibility (dashed line) increased in clear areas from 82% (A, black Analysis curve) to 89% (A, blue mate comparison curve), and in unclear areas increased from 20% (B, black Analysis curve) to 32% (B, blue mate comparison curve). On mated pairs, the percentage of minutiae marked by all examiners (unanimously marked) increased from 23% to 38% in median Clear areas (A, compare black Analysis and blue mate comparison lines at reproducibility=100%). (Figure is reproduced in color in the web version of this article.)

The net effect on minutia reproducibility was to increase from the Analysis to Comparison phase, but only for those latents compared to mated exemplars (not for those compared to nonmated exemplars). Fig. 19 shows this effect on a subset of 19 latents, each of which was assigned in both mated and nonmated image pairs; this subset controls for any differences in how latents were selected for the mated and nonmated pairs. Minutia reproducibility for mated pairs increased in both clear and unclear areas, which is generally representative of what was observed across all latents. For further discussion of how changes in markup relate to whether or not the exemplar was mated, see [7].

## 10 Corresponding minutiae

### 10.1 Probability of correspondence

**Fig. 20:**
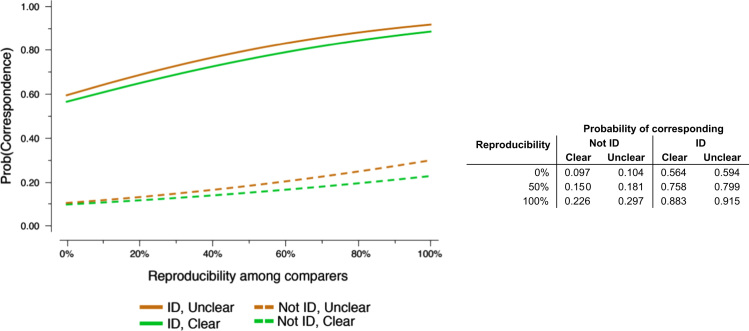
Probability of an examiner corresponding a minutia given the Comparison-phase reproducibility of that minutia among examiners who compared each image pair, conditioned on whether that examiner individualized, and whether that examiner said the minutia was clear. Probabilities calculated using logistic regression. (n=45,130 Comparison-phase minutiae; data from 11 latents that were each compared by only one examiner are excluded).

The probability of examiners corresponding marked minutiae was correlated with the reproducibility of those minutiae. Fig. 20 shows the probability of examiners corresponding minutiae as estimated by four logistic regression models, one for each combination of clarity (as marked by that examiner) and whether the examiner individualized.

### 10.2 Reproducibility of corresponding minutiae

In our previous work [2], we noted “Disagreements on sufficiency for individualization tend to be associated with substantial disagreements on corresponding minutiae.” Table 17, Table 18, Table 19, Table 20 describe reproducibility by type of correspondence markup as conditional probabilities: when examiner A marked a minutia, what did examiner B do? Table 17 summarizes reproducibility across all data; Table 18 through Table 20 summarize reproducibility on subsets of the data. The probabilities are calculated as weighted sums over all other examiners who marked each latent, such that each minutia marked by examiner A is weighted equally. The final column, “Marked and compared minutiae that were definitely corresponded,” is the probability that examiner B definitely corresponded a minutia given that examiner B marked that minutia and compared the latent to the exemplar. For example, Table 17 shows that when examiners corresponded minutiae marked as clear, 68.8% of the time other examiners also corresponded those minutiae; 20.0% of the time other examiners did not mark those minutiae at all. The data in these tables is limited to 3618 markups as described in Section 1.4.

Table 17 shows the very substantial interexaminer differences as to which minutiae were marked. Often when one examiner said a latent was NV, other examiners corresponded minutiae on that latent (recall that fingerprint comparisons in this test were selected to be borderline value). In addition to marking “definite” correspondences, examiners were instructed to indicate discrepancies (features in one print that definitely do not exist in the other print) as needed to support an exclusion determination. Examiners were also permitted to mark “debatable” correspondences: features “that potentially correspond, but do not meet your threshold for supporting an ID.” The correspondences referred to in [1] include only “definite” correspondences.

Whereas definite correspondences occurred much more often in clear than unclear areas (3x), debatable correspondences occurred about equally in clear and unclear areas. After controlling for clarity, minutiae that were marked as debatable correspondences have a similar, but slightly lower, reproducibility distribution to all minutiae.

Similar to the preceding tables, Table 21, Table 22 describe reproducibility by type of correspondence markup and whether the examiners changed their Analysis markup during Comparison. Table 23

**Fig. 21:**
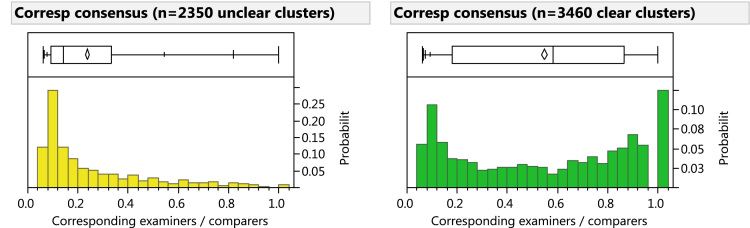
Consensus on whether to correspond clusters by clarity, among examiners who ***compared*** each image pair. For each cluster, consensus is measured as (number of examiners who corresponded at least one marked minutia in the cluster) / (number examiners who compared). Excludes 5 image pairs that were compared by fewer than three examiners; also excludes clusters that no examiner corresponded. (3,126 comparisons of 263 image pairs, 215 mated).

**Fig. 22:**
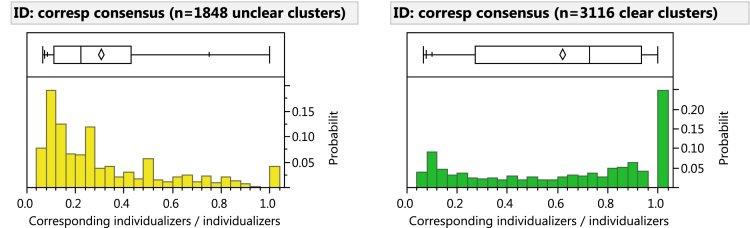
Consensus on whether to correspond clusters by clarity, among examiners who ***individualized*** each image pair. For each cluster, consensus is measured as (number of individualizing examiners who corresponded at least one marked minutia in the cluster) /(number examiners who individualized). Excludes 140 image pairs that were individualized by fewer than three examiners (60/231 mated pairs excluded); also excludes clusters that no individualizer corresponded. (1662 comparisons).

Fig. 21 shows the distribution of the proportion of examiners who corresponded each cluster by clarity among examiners who compared each image pair; Fig. 22 shows similar data limited to examiners who individualized the image pairs. These charts show that while consensus is generally low in unclear areas, consensus is mixed in clear areas: often a minority of examiners correspond minutiae in clear areas.

## 11 Reproducibility of minutia with respect to exclusion determinations

Responses included 561 exclusions on 81 mated and 75 nonmated pairs. When examiners determined that the latent and exemplar were not from the same source, they were asked to indicate a reason for the exclusion. Table 24 summarizes the distribution of reasons given. The distributions were not substantially different for nonmated and mated pairs (true and false exclusions). For 80% of exclusions, the reason given was “one or more minutiae differ.”

There were 25 mated pairs and 70 nonmated pairs that more than one examiner excluded. Agreement on exclusion reasons was low (beyond chance). For example, the probability that examiner B said “minutiae differ” given that examiner A said “minutiae differ” was 67% for mated pairs and 48% for nonmated pairs (each image pair weighted equally).

When examiners said “minutiae differ,” discrepancies were not usually marked (34% of mates, 42% of nonmates, 40% overall). Agreement on discrepancies was greater than chance, but not substantially. There were 47 image pairs on which at least two examiners marked discrepancies.

Upon completing the examinations that resulted in exclusions, examiners had marked 1744 minutiae (in 1264 clusters) on mated latents, 123 (7.1%) as discrepant; and 4901 minutiae (in 1703 clusters) on nonmated latents, 425 (8.7%) as discrepant. As shown in Table 25, there were 18 clusters with 3 discrepancies marked and 8 clusters with 4 discrepancies marked on nonmated image pairs (vs. 7 and 1 predicted from simulations that randomly assigned the “discrepant” label throughout the minutiae at the average rates for mates and nonmates).

Table 26 describes agreement on marking of discrepancies. When discrepancies were marked, they were more likely to be in clusters marked by many examiners: this pattern largely reflects chance (more opportunities for some examiner to note a discrepancy).

## 12 Variation in minutia locations

In order to better understand the lack of reproducibility, we clustered minutiae marked on the exemplars and then looked to see how these exemplar clusters corresponded to latent clusters. We expected to find many examples of exemplar clusters whose corresponding minutiae on the latents had not been assigned to a single cluster because of variation in the precise location at which examiners marked minutiae in unclear areas on the latent.

Clustering was performed on the 3618 exemplar markups (Comparison phase) described in Section 1.4 using the same clustering procedures and parameters as were used for the latents (3). Although clustering was performed on all minutiae marked on the exemplars, our analyses of variation in minutia locations focused on a subset of those minutiae that examiners marked as corresponding. In defining this subset, an additional 60 markups were omitted because of documentation errors in how the correspondences were marked. Most of these omitted markups were initially identified on the basis of having abnormally high bending energy (a measure of the non-linear component of the relative distortion between the minutiae marked on the latent and exemplar) [11], [12]). Each of the omitted markups was manually reviewed and most were identified as having “crossed” correspondences that were clearly incorrect (and presumably inadvertent documentation errors).

13,397 clusters were constructed from the 41,071 minutiae on the 3618 markups; 27,159 of these minutiae were marked as corresponding (after omitting the documentation errors). The 27,159 corresponding minutiae were contained in 5470 clusters on the exemplars and corresponded to 5794 clusters on the latents.

Table 27 summarizes correspondences among latent and exemplar clusters. 15% (830/5470) of exemplar clusters were corresponded to more than one latent cluster; 9% (538/5794) of latent clusters were corresponded to more than one exemplar cluster. 31% (1672/5470) of exemplar clusters were corresponded to only one latent cluster simply because only one minutia within the cluster was corresponded; similarly, 35% (2015/5794) of latent clusters.

Just as most minutiae were marked in median clear areas, this variation in the location at which examiners marked minutiae was most often observed in median clear areas: although examiners could be confident in the presence of these minutiae, certain aspects of clarity can interfere more with determining the precise location of minutiae than with determining their presence or absence. Variation in location (together with the clustering criteria) accounts for most of the lack of one-to-one correspondence between latent and exemplar clusters; examples of incorrect alignment of the latent and exemplar were also noted.

## Figures and Tables

**Table 1:** Examiner determinations for the four examples shown in Fig. 2.

	**Number of examiners**								**Mating**
	**Assigned**	**Value**	**VEO**	**NV**	**Compared**	**ID**	**Inc**	**Excl**	
**A**	15	12	2	1	14	9	2	3	Mate
**B**	15	14	1	–	15	15	–	–	Mate
**C**	14	13	–	1	13	13	–	–	Mate
**D**	11	11	–	–	11	1	2	8	Nonmate

**Table 2:** Effects of varying DBSCAN reachability distance. Minutia reproducibility distributions after DBSCAN clustering: oversized clusters were not split. (*n*=46,205 minutiae).

		**0.25 mm (0.010”)**	**0.38 mm (0.015”)**	**0.76 mm (0.030”)**
**Median reproducibility**	Clear	86%	91%	100%
	Unclear	18%	27%	42%
**# Clusters**	Clear	6484	5174	3496
	Unclear	5874	5035	3711
**% Singleton clusters**	Clear	34%	23%	12%
	Unclear	67%	60%	49%
**% Singleton minutiae**	Clear	6%	3%	1%
	Unclear	34%	26%	17%

**Table 3:** Reproducibility and consensus by clarity (Analysis phase, *n*=44,941 minutiae; 10,324 clusters).

	**Minutiae**	**Mean reproducibility**	**Median reproducibility**	**Mean consensus**	**Median consensus**
**Examiner clarity**					
Unclear	32,159	46.9%	46.2%	N/A	N/A
Clear	12,782	69.7%	81.8%	N/A	N/A
**Median clarity**					
Unclear	33,846	29.8%	22.2%	19.0%	10.0%
Clear	11,095	74.1%	84.6%	51.8%	50.0%
**Voted clarity**					
0–10% clear	1543	10.8%	0.0%	18.4%	12.5%
10–20% clear	1780	23.3%	14.3%	29.9%	20.0%
20–30% clear	2419	26.9%	20.0%	33.1%	27.3%
30–40% clear	3022	33.3%	30.0%	39.0%	36.4%
40–50% clear	2866	44.8%	44.4%	49.4%	50.0%
50–60% clear	4297	54.4%	58.3%	58.3%	61.5%
60–70% clear	5003	63.0%	70.0%	66.1%	72.7%
70–80% clear	4755	68.8%	76.9%	71.4%	78.6%
80–90% clear	6675	77.7%	87.5%	79.7%	88.9%
90–100% clear	12,581	86.9%	92.3%	88.0%	92.9%
**Overall**	44,941	63.2%	75.0%	36.3%	20.0%

**Table 4:** Percentages of minutiae that were marked in clear areas, conditioned on the level of consensus. (Analysis phase, *n*=44,941 minutiae).

	**Examiner clear**	**Median clear**
Singleton	49.7%	32.8%
Minority	56.2%	47.7%
Majority	72.8%	82.5%
Supermajority	86.2%	97.6%

**Table 5:** “Perfect” agreement counts those Analysis-phase markups in which (1) all minutiae that the examiner marked in clear areas were in majority clusters and (2) the examiner marked in all majority clusters (in any clarity). The 90% and 75% agreement columns require that at least 90% (75%) of the minutia that the examiner marked in clear areas were in majority clusters and the examiner marked at least 90% (75%) of the majority clusters. Latents lacking any clear minutiae or majority clusters trivially satisfy both criteria for “perfect” agreement.

**Any clear minutiae**	**Any majority clusters**	**Markups**	**“Perfect” agreement**		**90% agreement**		**75% agreement**	
Yes	Yes	2897	230	(8%)	479	(17%)	1462	(50%)
	No	18	0	(0%)	0	(0%)	0	(0%)
No	Yes	691	194	(28%)	220	(32%)	365	(53%)
	No	124	124	(100%)	124	(100%)	124	(100%)
**Total**		**3730**	**548**	**(15%)**	**823**	**(22%)**	**1951**	**(52%)**

**Table 6:** Distribution of singletons per markup (Analysis phase, mean of 12 examiners per latent).

**Category**	**Markups**	**Singletons**	**% markups**	**% singletons**
No singletons	1883	0	50	0
1 or 2 singletons	1299	1761	35	41
>2 singletons	548	2508	15	59
**Total**	**3730**	**4269**	**100**	**100**

**Table 7:** Probability of marking minutiae conditioned on the examiner׳s value assessment (Analysis phase, *n*=10,324 clusters).

**Consensus**	**P (marking|NV)**	**P (marking|VEO)**	**P (marking|VID)**
0.1	0.049	0.071	0.122
0.5	0.323	0.412	0.560
0.9	0.814	0.865	0.921

**Table 8:** Mean and median reproducibility of minutiae by ***examiner clarity*** and latent value assessment (Analysis phase, *n*=44,941 minutiae).

	**Mean reproducibility**			**Median reproducibility**		
	**Clear**	**Unclear**	**Overall**	**Clear**	**Unclear**	**Overall**
All	0.697	0.469	0.632	0.818	0.462	0.750
VID	0.705	0.469	0.646	0.833	0.462	0.750
VEO	0.614	0.450	0.541	0.733	0.455	0.600
NV	0.655	0.490	0.568	0.750	0.500	0.636

**Table 9:** Mean and median reproducibility of minutiae by ***median clarity*** and latent value assessment (Analysis phase, *n*=44,941 minutiae).

	**Mean reproducibility**			**Median reproducibility**		
	**Clear**	**Unclear**	**Overall**	**Clear**	**Unclear**	**Overall**
All	0.741	0.298	0.632	0.846	0.222	0.750
VID	0.743	0.287	0.646	0.846	0.214	0.750
VEO	0.725	0.304	0.541	0.833	0.222	0.600
NV	0.742	0.369	0.568	0.846	0.357	0.636

**Table 10:** Prevalence of nonminutia features in the area of minutia clusters (Comparison phase, *n*=10,398 clusters). Here we consider a nonminutia feature as being in a minutia cluster if it is within 0.38 mm (0.015”) of the cluster center. We report Comparison-phase counts because examiners were only instructed to mark “other” features during Comparison.

	**Features**	**Features in clusters**		**Clusters with nonminutia features**	
Cores	1269	519	40.9%	174	1.7%
Deltas	621	180	29.0%	78	0.8%
Other nonminutia features	703	320	45.5%	223	2.1%
Total nonminutia features	2593	1019	39.3%	465	4.5%

**Table 11:** Examiner B clarity by examiner A clarity for each ***minutia*** marked by examiner A. Data is constructed from all pairs of examiners on each latent; each minutia marked by examiner A is equally weighted (Analysis phase, *n*=44,941 minutiae). The tables summarize the clarity examiner B assigned to each location without regard to whether examiner B marked a minutia at that location.

***Minutiae***			Examiner B						Total minutiae
			Unclear			Clear			
			Black	Red	Yellow	Green	Blue	Aqua	
Examiner A	Unclear	Black	60	55	206	434	87	22	863
		Red	41	158	447	357	49	5	1056
		Yellow	324	1026	4258	4505	653	93	10,859
	Clear	Green	656	956	5858	14,608	3111	565	25,754
		Blue	119	86	701	3060	1085	220	5271
		Aqua	35	9	102	569	222	201	1138



***Minutiae***		Examiner B		Total minutiae
		Unclear	Clear	
Examiner A	Unclear	6574 (51%)	6204 (49%)	12,778
	Clear	8522 (26%)	23,641 (74%)	32,163

**Table 12:** Examiner B clarity by examiner A clarity at each ***cluster*** center. Data is constructed from all pairs of examiners on each latent regardless of whether the examiners marked in the cluster; each cluster is weighted equally (*n*=10,324 clusters). The tables summarize the clarity examiners assigned to each cluster without regard to whether those examiners marked a minutia in the cluster.

***Clusters***			Examiner B						Total clusters
			Unclear			Clear			
			Black	Red	Yellow	Green	Blue	Aqua	
Examiner A	Unclear	Black	86	57	127	124	21	5	420
		Red	57	238	484	233	25	2	1039
		Yellow	127	484	1648	1228	150	19	3657
	Clear	Green	124	233	1228	2216	418	71	4292
		Blue	21	25	150	418	129	26	770
		Aqua	5	2	19	71	26	23	147



***Clusters***		Examiner B		Total clusters
		Unclear	Clear	
Examiner A	Unclear	3308	1808	5116
		(65%)	(35%)	
	Clear	1808	3400	5208
		(35%)	(65%)	

**Table 13:** Examiner B clarity by examiner A clarity for each minutia marked by examiner A, conditioned by whether examiner B marked a minutia at that location. Data constructed from all pairs of examiners on each latent; each minutia marked by examiner A is equally weighted (*n*=44,941 Analysis-phase minutiae).

***Minutiae***		B marked			B not marked			Total minutiae
		Unclear	Clear	Subtotal	Unclear	Clear	Subtotal	
Examiner A	Unclear	2127	4014	6141	4384	2253	6637	12,778
		(35%)	(65%)		(66%)	(34%)		
	Clear	4016	18,590	22,606	4448	5109	9557	32,163
		(18%)	(82%)		(47%)	(53%)		

**Table 14:** Percentage of minutiae that are “relatively far” (more than 0.1”, about 5 ridge intervals on average) or “very far” (more than 0.2”, about 10 ridge intervals) from the nearest majority cluster, by phase and minutia clarity. The total minutia count is limited to latents that had at least one majority cluster. For corresponding minutiae, distance is measured to the nearest cluster that was marked and corresponded by a majority of comparing examiners. (Analysis phase, *n*=44,729; another 212 minutiae were marked on latents having no majority clusters).

		**Minutiae**	**Relatively far**		**Very far**	
			**(Distance >0.1”)**		**(Distance >0.2”)**	
			**Minutiae**	**%**	**Minutiae**	**%**
Marked minutiae (Analysis phase)	Total	44,729	5006	11.2	1581	3.5%
	Examiner Clear	32,081	2250	7.0	701	2.2%
	Examiner Unclear	12,648	2756	21.8	880	7.0%
	Median Clear	33,840	1094	3.2	176	0.5%
	Median Unclear	10,889	3912	35.9	1405	12.9%
Corresponding minutiae (Comparison phase)	Total	27,486	2277	8.3	632	2.3%
	Examiner Clear	20,271	1110	5.5	317	1.6%
	Examiner Unclear	7215	1167	16.2	315	4.4%

**Table 15:** Reproducibility of ***Analysis*** minutiae by clarity and change type (n=42,279 Analysis-phase minutiae). Data are limited to 3709 responses on 320 image pairs, which excludes 31 markups with data collection problems (detailed in [7]).

**Clarity**	**Reproducibility**	**Retained**	**Moved**	**Deleted**	**% Deleted**
Clear	SuperMajority	11,953	701	236	1.8%
	Majority	9555	667	475	4.4%
	Minority	4274	361	646	12.2%
	Singleton	1410	108	515	25.3%
Unclear	SuperMajority	1707	132	53	2.8%
	Majority	3201	261	207	5.6%
	Minority	3203	230	448	11.5%
	Singleton	1439	82	415	21.4%
All	SuperMajority	13,660	833	289	2.0%
	Majority	12,756	928	682	4.7%
	Minority	7477	591	1094	11.9%
	Singleton	2849	190	930	23.4%

**Table 16:** Reproducibility of ***Comparison*** minutiae by clarity and change type (*n*=46,119 Comparison-phase minutiae). Data are limited to 2957 comparisons of 313 image pairs, which excludes markups where either the latent or exemplar was assessed to be NV and some data collection problems (detailed in [7]).

**Clarity**	**ReproCategory**	**Retained**	**Moved**	**Added**	**% Added**
Clear	SuperMajority	12,675	714	768	5.4%
	Majority	9095	686	1449	12.9%
	Minority	3966	303	1229	22.4%
	Singleton	1346	100	506	25.9%
Unclear	SuperMajority	1590	157	237	11.9%
	Majority	3198	289	933	21.1%
	Minority	3031	209	1380	29.9%
	Singleton	1443	73	742	32.9%
All	SuperMajority	14,265	871	1005	6.2%
	Majority	12,293	975	2382	15.2%
	Minority	6997	512	2609	25.8%
	Singleton	2789	173	1248	29.6%

**Table 17:** When examiner A marked a minutia, what examiner B did (*n*=50,894 minutiae marked during Analysis or added during Comparison). Without regard to clarity, 63.1% of the minutiae definitely corresponded by examiner A were also definitely corresponded by examiner B; 10.9% of examiner A׳s discrepancies were definitely corresponded by examiner B.

***ALL Minutiae***				Minutiae	**Examiner B**						Marked and compared minutiae that were definitely corresponded
					Did not mark	Marked					
						Not Compared (NV)	Compared				
							Not corresponded		Corresponded		
							Unassoc.	Discrepant	Debatable	Definite	
**Examiner A**	**Clear**	NV		1379	33.4%	25.0%	20.2%	1.0%	1.7%	18.7%	45.0%
		Not corresponded	Unassoc.	12,231	36.8%	2.8%	43.7%	1.5%	1.4%	13.8%	22.9%
			Discrepant	457	32.7%	4.2%	41.9%	6.9%	1.6%	12.7%	20.2%
		Corresponded	Debatable	677	36.6%	4.2%	23.4%	1.0%	3.7%	30.9%	52.3%
			Definite	20,470	20.0%	1.5%	8.2%	0.3%	1.3%	68.8%	87.6%
	**Unclear**	NV		1447	49.7%	19.5%	16.8%	0.8%	1.4%	11.8%	38.4%
		Not corresponded	Unassoc.	5844	60.4%	3.0%	25.3%	0.9%	1.2%	9.2%	25.0%
			Discrepant	175	56.5%	3.4%	27.4%	5.4%	1.2%	6.0%	15.1%
		Corresponded	Debatable	755	63.2%	2.0%	10.2%	0.3%	2.3%	22.0%	63.3%
			Definite	7459	42.1%	1.8%	7.1%	0.2%	1.6%	47.3%	84.2%

**Table 18:** When examiner A marked a minutia, what examiner B did, limited to minutiae marked on mated pairs. Without regard to clarity, 63.7% of the minutiae definitely corresponded by examiner A were also definitely corresponded by examiner B.

***Mates***				Minutiae	**Examiner B**						Marked and compared minutiae that were definitely corresponded
					Did not mark	Marked					
						Not Compared (NV)	Compared				
							Not corresponded		Corresponded		
							Unassoc.	Discrepant	Debatable	Definite	
**Examiner A**	**Clear**	NV		937	32.5%	23.2%	16.0%	0.1%	1.9%	26.3%	59.4%
		Not corresponded	Unassoc.	8613	38.6%	2.3%	38.9%	0.3%	1.4%	18.5%	31.2%
			Discrepant	137	34.8%	1.4%	24.0%	1.2%	1.3%	37.3%	58.4%
		Corresponded	Debatable	575	38.6%	3.8%	19.4%	0.2%	3.2%	35.0%	60.7%
			Definite	20,245	19.8%	1.4%	7.8%	0.2%	1.2%	69.5%	88.2%
	**Unclear**	NV		1013	48.7%	18.9%	14.3%	0.2%	1.4%	16.4%	50.7%
		Not corresponded	Unassoc.	4189	62.0%	2.4%	22.1%	0.2%	1.2%	12.1%	34.0%
			Discrepant	48	68.8%	1.7%	13.3%	0.9%	0.5%	14.8%	50.0%
		Corresponded	Debatable	672	63.6%	1.6%	8.2%	0.2%	2.1%	24.3%	70.0%
			Definite	7391	42.0%	1.7%	6.9%	0.2%	1.5%	47.6%	84.7%

**Table 19:** When examiner A marked a minutia, what examiner B did, limited to minutiae marked on nonmated pairs. Without regard to clarity, 8.1% of the minutiae definitely corresponded by examiner A were also definitely corresponded by examiner B.

***Nonmates***				Minutiae	**Examiner B**						Marked and compared minutiae that were definitely corresponded
					Did not mark	Marked					
						Not Compared (NV)	Compared				
							Not corresponded		Corresponded		
							Unassoc.	Discrepant	Debatable	Definite	
**Examiner A**	**Clear**	NV		442	35.4%	28.9%	29.0%	3.0%	1.2%	2.5%	7.1%
		Not corresponded	Unassoc.	3618	32.4%	4.2%	55.0%	4.3%	1.3%	2.8%	4.4%
			Discrepant	320	31.7%	5.4%	49.6%	9.4%	1.7%	2.2%	3.6%
		Corresponded	Debatable	102	25.9%	6.9%	46.5%	5.9%	7.0%	7.9%	11.8%
			Definite	225	31.6%	5.8%	46.9%	3.9%	3.8%	8.0%	12.8%
	**Unclear**	NV		434	51.9%	20.8%	22.6%	2.1%	1.4%	1.2%	4.4%
		Not corresponded	Unassoc.	1655	56.5%	4.5%	33.2%	2.7%	1.3%	1.7%	4.4%
			Discrepant	127	51.9%	4.0%	32.8%	7.1%	1.5%	2.7%	6.2%
		Corresponded	Debatable	83	59.5%	5.4%	26.4%	1.5%	3.9%	3.3%	9.4%
			Definite	68	45.6%	4.8%	35.2%	2.9%	3.3%	8.3%	16.7%

**Table 20:** When examiner A marked a minutia, what examiner B did, limited to minutiae marked when both examiners individualized; based on 185 image pairs that were individualized by at least two examiners (out of 231 mated pairs). Without regard to clarity, 69.4% of the minutiae definitely corresponded by examiner A were also definitely corresponded by examiner B.

***Both ID***				Minutiae	**Examiner B**						Marked and compared minutiae that were definitely corresponded
					Did not mark	Marked					
						Not Compared (NV)	Compared				
							Not corresponded		Corresponded		
							Unassoc.	Discrepant	Debatable	Definite	
**Examiner A**	**Clear**	NV		N/A							
		Not corresponded	Unassoc.	5125	39.7%	N/A	38.5%	0.1%	1.2%	20.5%	34.0%
			Discrepant	8	48.1%	N/A	50.9%	0.0%	0.0%	1.0%	1.9%
		Corresponded	Debatable	317	35.1%	N/A	18.8%	0.0%	2.9%	43.2%	66.5%
			Definite	18,738	17.3%	N/A	5.5%	0.0%	0.9%	76.4%	92.4%
	**Unclear**	NV		N/A							
		Not corresponded	Unassoc.	2228	63.6%	N/A	20.8%	0.0%	0.9%	14.7%	40.5%
			Discrepant	7	83.3%	N/A	16.7%	0.0%	0.0%	0.0%	0.0%
		Corresponded	Debatable	356	62.8%	N/A	6.3%	0.0%	1.7%	29.2%	78.5%
			Definite	6558	36.6%	N/A	5.2%	0.0%	1.2%	57.0%	89.9%

**Table 21:** When examiner A marked a minutia, what examiner B did (n=50,894 minutiae marked during Analysis or added during Comparison).

***ALL Minutiae***			Minutiae	**Examiner B**							Marked and compared minutiae that were definitely corresponded
				Did not mark	Marked						
					NV (Not Compared)	Compared					
						Not corresponded				Definite Corresp.	
						Retained	Moved	Deleted	Added		
**Examiner A**	NV		2826	41.8%	22.2%	17.6%	0.8%	1.6%	0.8%	15.2%	42.1%
	Not corresponded	Retained	15,384	39.4%	3.2%	41.2%	0.8%	2.2%	1.1%	12.0%	21.0%
		Moved	440	40.1%	5.2%	27.2%	1.3%	3.5%	1.2%	21.6%	39.5%
		Deleted	2895	63.4%	1.6%	12.0%	0.5%	5.3%	0.8%	16.4%	46.8%
		Added	1420	65.4%	1.6%	11.7%	0.4%	1.5%	1.5%	17.8%	54.1%
	Corresponded		27,929	25.9%	1.5%	6.6%	0.3%	1.7%	0.9%	63.1%	86.9%

**Table 22:** When examiner A marked a minutia, what examiner B did (n=35,214 minutiae marked by examiner A as Clear during Analysis or added during Comparison).

***CLEAR Minutiae***			Minutiae	**Examiner B**							Marked and compared minutiae that were definitely corresponded
				Did not mark	Marked						
					NV (Not Compared)	Compared					
						Not corresponded				Definite Corresp.	
						Retained	Moved	Deleted	Added		
**Examiner A**	NV		1379	33.4%	25.0%	19.6%	0.7%	1.7%	0.9%	18.7%	45.0%
	Not corresponded	Retained	10,624	31.8%	3.1%	47.5%	0.8%	2.2%	1.1%	13.4%	20.6%
		Moved	307	36.3%	5.5%	30.4%	1.4%	3.7%	1.2%	21.5%	36.9%
		Deleted	1810	58.6%	1.8%	13.8%	0.6%	5.5%	0.8%	18.9%	47.8%
		Added	624	56.2%	2.1%	18.1%	0.4%	1.7%	1.4%	20.1%	48.2%
	Corresponded		20,470	20.0%	1.5%	7.1%	0.4%	1.6%	0.8%	68.8%	87.6%

**Table 23:** (A) Cluster clarity by consensus on whether to correspond minutiae, among examiners who ***compared*** each image pair (same data as Fig. 21; n=5810 clusters); (B) Cluster clarity by consensus on whether to correspond minutiae, among examiners who ***individualized*** each image pair (same data as Fig. 22; *n*=4975 clusters).

**A) Compared**	**Unclear**		**Clear**		**Total clusters**
	**Clusters**	**%**	**Clusters**	**%**	
Singleton	990	68%	460	32%	1450
Minority	1037	49%	1058	51%	2095
Majority	297	21%	1119	79%	1416
SuperMajority	26	3%	823	97%	849


**B) ID**	**Unclear**		**Clear**		**Total clusters**
	**Clusters**	**%**	**Clusters**	**%**	
Singleton	753	65%	398	35%	1151
Minority	667	48%	720	52%	1387
Majority	347	27%	958	73%	1305
SuperMajority	81	7%	1040	93%	1121


**Table 24:** Exclusion reasons. Examiners were instructed to select the first option that applied. The exclusion reason was missing for one comparison.

**Exclusion reason**	**Mates**		**Nonmates**	
Pattern classes differ	12	9%	49	9%
Core or delta differences	8	6%	50	10%
One or more minutiae differ	104	80%	447	80%
Level-3 features differ	3	2%	6	1%
Other	3	2%	8	1%
**Total**	**130**	**100%**	**430**	**100%**


**Table 25:** Counts of discrepant minutiae among clusters on exclusion determinations by whether the cluster was a singleton. For example, 97 clusters on mated pairs that were marked by more than one examiner (“Not singleton”) were marked as discrepant by exactly one examiner. In no case did more than four examiners mark a minutia as discrepant.

	**Mates**					**Nonmates**					
	**Number of discrepancies**					**Number of discrepancies**					
	**0**	**1**	**2**	**3**	**Total**	**0**	**1**	**2**	**3**	**4**	**Total**
Singleton	252	17	0	0	269	663	48	0	0	0	711
Not singleton	894	97	3	1	995	714	212	40	18	8	992
**Total clusters**	**1146**	**114**	**3**	**1**	**1264**	**1377**	**260**	**40**	**18**	**8**	**1703**


**Table 26:** Percentage of clusters marked as discrepant by any comparing examiner by Comparison-phase consensus.

	**Mates**			**Nonmates**		
	**Clusters**	**Discrepancies**	**% Discrep**	**Clusters**	**Discrepancies**	**% Discrep**
Singleton	269	17	6%	711	48	7%
Minority	252	25	10%	354	72	20%
Majority	365	43	12%	406	178	44%
SuperMajority	378	38	10%	232	128	55%
**Total**	**1264**	**123**	**10%**	**1703**	**426**	**25%**


**Table 27:** Correspondences among latent and exemplar clusters.

	**Latent clusters**	**Exemplar clusters**
Only one minutia in the cluster was corresponded	2015	1672
More than one minutia in the cluster was corresponded	3779	3798
*those minutiae corresponded to the same cluster*	*3241*	*2968*
*those minutiae corresponded to different clusters*	*538*	*830*
**Total**	**5794**	**5470**


## Footnotes

1 The DBSCAN MATLAB source code was downloaded from http://www.chemometria.us.edu.pl/index.php?goto=downloads

2 MATLAB version R2014a. MATLAB׳s implementation of agglomerative hierarchical clustering is documented at www.mathworks.com/help/stats/linkage.html.
