# Supplementary material for: Data on the interexaminer variation of minutia markup on latent fingerprints
Source: Data Brief. 2016 May 4;8:158–90. doi: 10.1016/j.dib.2016.04.068 (PMC4889892; doi:10.1016/j.dib.2016.04.068)
Supplement: Supplementary file 1 — Supplementary material [file mmc1.docx]

***Conflicts of Interest Statement***

**Manuscript title:**

Data on the interexaminer variation of minutia markup on latent fingerprints

The authors whose names are listed immediately below certify that they have NO affiliations with or involvement in any organization or entity with any financial interest (such as honoraria; educational grants; participation in speakers’ bureaus; membership, employment, consultancies, stock ownership, or other equity interest; and expert testimony or patent-licensing arrangements), or non-financial interest (such as personal or professional relationships, affiliations, knowledge or beliefs) in the subject matter or materials discussed in this manuscript.

**Author names:**

N/A

The authors whose names are listed immediately below report the following details of affiliation or involvement in an organization or entity with a financial or non-financial interest in the subject matter or materials discussed in this manuscript. Please specify the nature of the conflict on a separate sheet of paper if the space below is inadequate.

**Author names:**

Bradford T. Ulery – employee of Noblis; contractor to Federal Bureau of Investigation (US Government)

R. Austin Hicklin – employee of Noblis; contractor to Federal Bureau of Investigation (US Government)

Maria Antonia Roberts – employee of Federal Bureau of Investigation (US Government)

JoAnn Buscaglia – employee of Federal Bureau of Investigation (US Government)

**
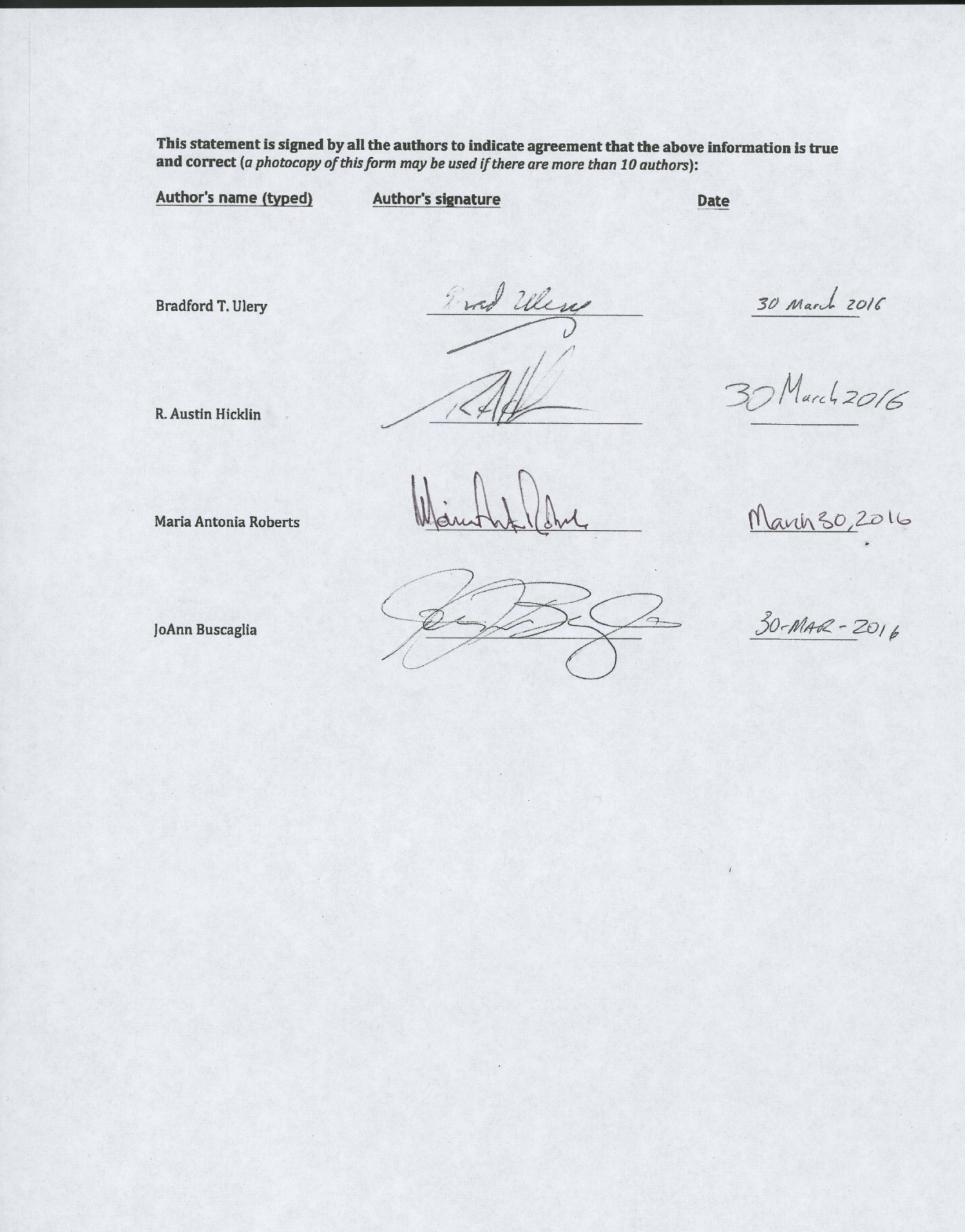
**
